# Identification and external validation of a prognostic signature based on hypoxia–glycolysis-related genes for kidney renal clear cell carcinoma

**DOI:** 10.1515/med-2025-1305

**Published:** 2025-10-31

**Authors:** Zijian Zhou, Yuan Xiang, Annan Wu, Yiwei Zhang, Lei Xie, Yajie Zhou, Wenxiong Zhang, Queling Liu

**Affiliations:** Department of Thoracic Surgery, The Second Affiliated Hospital, Jiangxi Medical College, Nanchang University, 1 Minde Road, Donghu District, Nanchang, 330006, China; The Second Clinical Medical School, Jiangxi Medical College, Nanchang University, Nanchang, 330088, China; Department of Urology, The Second Affiliated Hospital, Jiangxi Medical College, Nanchang University, Nanchang, 330006, China; Queen Mary School, Jiangxi Medical College, Nanchang University, Nanchang, 330088, China; Department of Oncology, The Second Affiliated Hospital, Jiangxi Medical College, Nanchang University, 1 Minde Road, Donghu District, Nanchang, 330006, China

**Keywords:** prognostic signature, hypoxia, glycolysis, kidney renal clear cell carcinoma

## Abstract

**Background:**

Hypoxia and glycolysis play crucial roles in tumor progression, yet their association with kidney renal clear cell carcinoma (KIRC) remains unclear. Here, a novel prognostic model was developed with hypoxia–glycolysis-related genes (HGRGs) in KIRC, providing insights to elucidate the aforementioned uncertainties.

**Methods:**

Transcriptomic information and clinical characteristics of KIRC were acquired from The Cancer Genome Atlas Program, ArrayExpress database, and Gene Expression Omnibus. Significant HGRGs were identified, and a prognostic model was constructed. We performed enrichment analysis, tumor mutational burden (TMB), tumor microenvironment), and drug sensitivity analyses to elucidate potential mechanisms of HGRGs.

**Results:**

The prognostic model based on five HGRGs (ADORA2B, TGFA, FBP1, HK3, PDHB) effectively predicted the clinical outcome. The nomogram, which integrates a prognostic model and clinical information, demonstrated superior performance. Low-risk patients were enriched in fatty acid metabolism and peroxisome pathways, exhibited higher immunotherapy responsiveness, and showed greater sensitivity to Gefitinib and Afatinib. High-risk patients exhibited activation of inflammatory and profibrotic pathways, an elevated TMB, immunosuppressive microenvironments, and greater sensitivity to Topotecan and Irinotecan. RT-qPCR validated the expression of HGRGs across selected cell lines.

**Conclusions:**

The prognostic model derived from five HGRGs demonstrates excellent clinical value in predicting prognosis and guiding therapeutic strategies in KIRC.

## 1 Introduction

Renal cell carcinoma (RCC) is a significant contributor to mortality among urinary system tumors, with kidney renal clear cell carcinoma (KIRC) representing the dominant pathological variant in RCC. Globally, over 400,000 new diagnoses and 140,000 fatal outcomes of KIRC are documented yearly [1,2]. Widely adopted in clinical practice, the tumor–node–metastasis (TNM) classification method remains the gold standard for staging tumors and assessing prognosis [3]. However, the TNM classification system often fails to meet the clinical requirements [4]. The advent of biomarker-based prognostic models has provided fresh insights into the prognostic assessment of patients with tumors. Consequently, the discovery of emerging biomarkers and the establishment of new prognostic models are urgently required.

Hypoxia, a critical hallmark of cancer, is present in nearly 90% of solid tumors [5]. Under hypoxic conditions, tumor cells, confronted with limited oxygen and energy, can rely on glycolysis to generate the energy required for proliferation [6,7]. Recent studies have shown that a prognostic model based on hypoxia or glycolysis separately can effectively predict the clinical outcomes of kidney cancer. Ning et al. constructed a hypoxia-related gene (HRG) model in KIRC to accurately predict the survival outcome of endometrial cancer patients and guide therapeutic strategies [8]. A glycolysis-related prognostic model based on ten signatures constructed by Xing et al. successfully predicts the survival outcome of KIRC [9]. However, there have been limited studies on prognostic models integrating hypoxia and glycolysis in KIRC.

The primary objective of this research was to develop a predictive model based on hypoxia–glycolysis-related genes (HGRGs) using 101 algorithm combinations to forecast patient prognosis and to guide clinical practice in KIRC.

## 2 Materials and methods

### 2.1 Data sources

We retrieved the public sequencing information, encompassing raw counts and transcripts per million (TPM) from The Cancer Genome Atlas (TCGA)-KIRC, along with related clinical information. For the subsequent analysis, TPM data downloaded from the TCGA database were converted to log2(TPM + 1) units. Additionally, the ArrayExpress database provided E-MTAB-1980, which was employed in this study as an independent dataset for external validation purposes. To further enhance validation, we incorporated an additional independent cohort, GSE22541 from Gene Expression Omnibus (GEO), which contains 42 tumor samples. This study comprised 529 tumor specimens obtained from TCGA-KIRC and 71 normal samples, supplemented by 101 tumor cases from E-MTAB-1980 and 42 tumor samples from GSE22541. Selected samples fulfilled eligibility requirements and were subsequently subjected to the following comprehensive analytical workflows.

### 2.2 Identification of differentially expressed HGRGs

HRGs and glycolysis-related genes (GRGs) were sourced from the Molecular Signatures Database (MsigDB). We identified 354 HRGs linked to Harris hypoxia, GOBP regulation of cellular response to hypoxia, Reactome cellular response to hypoxia, and Hallmark hypoxia. Additionally, 310 GRGs were identified from Kyoto encyclopedia of genes and genome (KEGG) glycolysis gluconeogenesis, WP glycolysis and gluconeogenesis, Reactome glycolysis, and Hallmark glycolysis in the MsigDB database. Differentially expressed genes (DEGs) were statistically defined through R (version 4.3.3) and the DESeq2 package with a cutoff of false discovery rate (FDR) < 0.05 and |log2FoldChange| > 1 [10]. We then intersected the DEGs with HRGs and GRGs, ultimately identifying 52 HGRGs.

### 2.3 Development of a machine learning-driven prognostic model integrating hypoxia and glycolysis genes

TCGA-KIRC patients were randomly assigned to training and testing cohorts with the same sample size. The E-MTAB-1980 cohort was positioned as a verification group for further validation. The detailed clinical characteristics of the TCGA-KIRC training and testing cohorts, E-MTAB-1980, and GSE22541 are listed in Table 1. Initially, we validated the prognostic potential of 25 out of 52 candidate HGRGs through univariate Cox analysis. Subsequently, a comprehensive analysis was conducted using ten diverse algorithms – Ridge, Stepwise Cox, Lasso, CoxBoost, Enet, plsCox, SuperPC, RSF, GBM, and SVM – along with 101 algorithm combinations. For each combination, we systematically evaluated model performance using Harrell’s C-index on all combinations. The ultimate prediction model was constructed by using the highest-performing algorithm that achieved the maximal C-index [11].

**Table 1:** Clinical characteristics of TCGA-KIRC, E-MTAB-1980, and GSE22541

Characteristics	TCGA train cohort (*n* = 265)		TCGA test cohort (*n* = 264)		E-MTAB-1980 cohort (*n* = 101)		GSE22541 cohort (*n* = 42)	
	*n*	Percentage	*n*	Percentage	*n*	Percentage	*n*	Percentage
**Age**								
<= 65	180	67.92	168	63.64	57	56.44	—	—
>65	85	32.08	96	36.36	44	43.56	—	—
**Status**								
Alive	185	69.81	171	64.77	78	77.23	31	73.81
Dead	80	30.19	93	35.23	23	22.77	11	26.19
**Gender**								
Female	97	36.60	88	33.33	24	23.76	16	38.10
Male	168	63.40	176	66.67	77	76.24	26	61.90
**Grade**								
G1	9	3.40	4	1.52	—	—	—	—
G2	118	44.53	110	41.67	—	—	—	—
G3	104	39.25	101	38.26	—	—	—	—
G4	30	11.32	45	17.05	—	—	—	—
Unknown	4	1.51	4	1.52	—	—	—	—
**Stage**								
Stage I	138	52.08	125	47.35	—	—	—	—
Stage II	25	9.43	32	12.12	—	—	—	—
Stage III	62	23.40	61	23.11	—	—	—	—
Stage IV	39	14.72	44	16.67	—	—	—	—
Unknown	1	0.38	2	0.76	—	—	—	—
**T stage**								
T1	141	53.21	128	48.48	68	67.34	—	—
T2	32	12.08	37	14.02	11	10.89	—	—
T3	86	32.45	94	35.61	21	20.79	—	—
T4	6	2.26	5	1.89	1	0.99	—	—
**M stage**								
M0	217	81.89	203	76.89	89	88.12	—	—
M1	38	14.34	41	15.53	12	11.88	—	—
Unknown	10	3.77	20	7.58	—	—	—	—
**N stage**								
N0	123	46.42	116	43.94	94	93.07	—	—
N1	7	2.64	9	3.41	3	2.97	—	—
N2	0	0.00	0	0.00	4	3.96	—	—
Unknown	135	50.94	139	52.65	0	0.00	—	—

Abbreviations: T stage: tumor stage; N stage: node stage; M stage: metastasis stage.

### 2.4 Hypoxia–glycolysis-related prognostic model

Risk scores were computed using the predictive model results based on the subsequent equation:

\[{\mathrm{Risk\; score}}=\mathop{\sum }\limits_{i=1}^{n}{\mathrm{coefficient}}\left({{\mathrm{gene}}}_{{\mathrm{i}}})\left\times {\mathrm{expression}}\left({{\mathrm{gene}}}_{i}).]\]

Employing the midpoint of the risk score value as a threshold, patients in TCGA-KIRC (including those in the training or testing sets), E-MTAB-1980, and GSE22541 were categorized into a high-risk group (HG) or a low-risk group (LG). Kaplan–Meier (K-M) survival analysis based on the R package “survival” was conducted between HG and LG in TCGA-KIRC and E-MTAB-1980 to evaluate overall survival. To assess the predictive value of our model across different cohorts, we constructed time-dependent receiver operating characteristic (ROC) curves based on the R package “timeROC” to predict survival. Principal component analysis (PCA) analysis was executed with the expressions of TCGA-KIRC along with E-MTAB-1980 to assess subgroup-related effects in the prognostic model. Univariate and multivariate Cox regression analyses were executed to measure independent prognostic value of our model. Finally, we applied the K-M survival analysis to investigate the model’s practical utility across various clinical traits.

### 2.5 Construction of a nomogram for prognosticating clinical outcomes

We developed a nomogram that comprised age, gender, stage, and risk model as predictive factors for forecasting the clinical outcome in KIRC patients, using the R package “rms” [12]. To determine clinical utility, decision curve analysis (DCA) and ROC curves were implemented to contrast the nomogram’s forecasting ability with conventional clinical information. Additionally, calibration curves were employed to quantitatively compare the nomogram incorporating the prognostic model with the nomogram without the prognostic model.

### 2.6 Enrichment analysis

The study implemented KEGG and gene ontology (GO) analyses to uncover the potential functional roles associated with the HGRGs by utilizing the “clusterProfiler” R package [13]. Based on the risk groups defined by the prognostic model, gene set enrichment analysis (GSEA) was conducted by applying GSEA_4.3.2 software to explore significant pathways, with cutoff criteria of *p* < 0.05 and FDR < 0.25 [14]. The results were visualized using the GseaVis package [15].

### 2.7 Tumor mutational burden (TMB)

Detailed somatic mutation data for KIRC were systematically acquired through querying and downloading processes performed on the TCGA platform and stratified into the HG and LG based on the previously established prognostic model. Mutation landscapes were visualized using waterfall diagrams generated by the “maftools” R package [16,17].

### 2.8 Tumor microenvironment (TME) characterization and immune infiltration

Comparative evaluation of TME characteristics was conducted using the “ESTIMATE” R package. Additional insights were derived from the Tumor Immune Estimation Resource portal. Furthermore, seven methods – QUANTISEQ, CIBERSORT-ABS, TIMER, EPIC, MCPCOUNTER, and XCELL – were applied to probe the associations between distinct immune cells and risk scores [18]. The Immuno-Oncology Biological Research (IOBR) R package was used to assess the immune features and immune cell infiltration in HG and LG [19]. Subsequently, single-sample GSEA for the comparison between immune function and different risk groups was conducted with the “GSVA” package.

### 2.9 HGRG in immunotherapy and chemotherapy

To stratify immune evasion patterns, we accessed tumor immune dysfunction and exclusion (TIDE) profiles for KIRC patients through the official TIDE web portal [20]. Drug sensitivity analysis was implemented via the “oncoPredict” R package, where drug-specific half-maximal inhibitory concentration (IC50) values were predicted and statistically compared between HG and LG patients following the established model [21].

### 2.10 Immunohistochemistry staining and RT-qPCR

In the Human Protein Atlas (HPA), we accessed immunohistochemical profiles of differentially expressed HGRGs across normal and cancer tissues. For this study, human normal kidney cell lines (HK-2) and two KIRC cell lines (CAKI-1 and 786-O) were accessed from Cobioer Biosciences (Nanjing, China). TRIzol reagent (Takara, Japan) was utilized to extract total RNA from cells. For cDNA synthesis, reagents from the reverse transcription kit by Accurate Biology (Hunan, China) were utilized. Quantitative PCR reactions were then executed on the LightCycler­® 480 Instrument II (Roche, Switzerland) using SYBR Green premixed qPCR kits supplied by Accurate Biology (Hunan, China). The endogenous reference gene β-actin was used to standardize expression values. Sample comparability was achieved by computing relative mRNA quantification via the 2^ΔΔCT^ methodology. The primer sequences for the five HGRGs used in RT-qPCR are listed in Table S1.


**Ethical approval:** This article does not contain any studies with human participants or animals performed by any of the authors.

## 3 Results

### 3.1 Screening for HGRGs in KIRC patients and enrichment analysis

**Figure 1:**
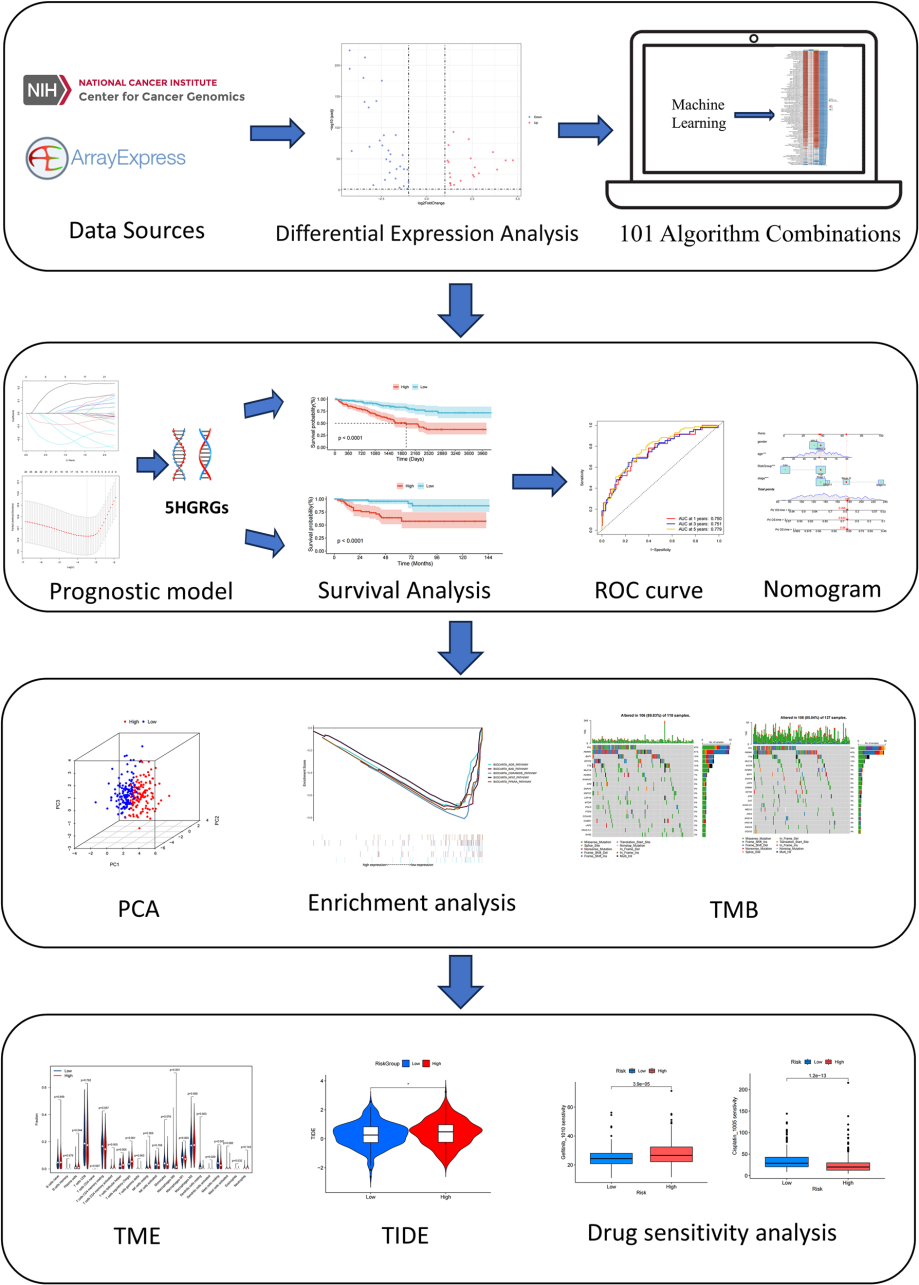
Schematic of the integrated experimental research workflow.

**Figure 2:**
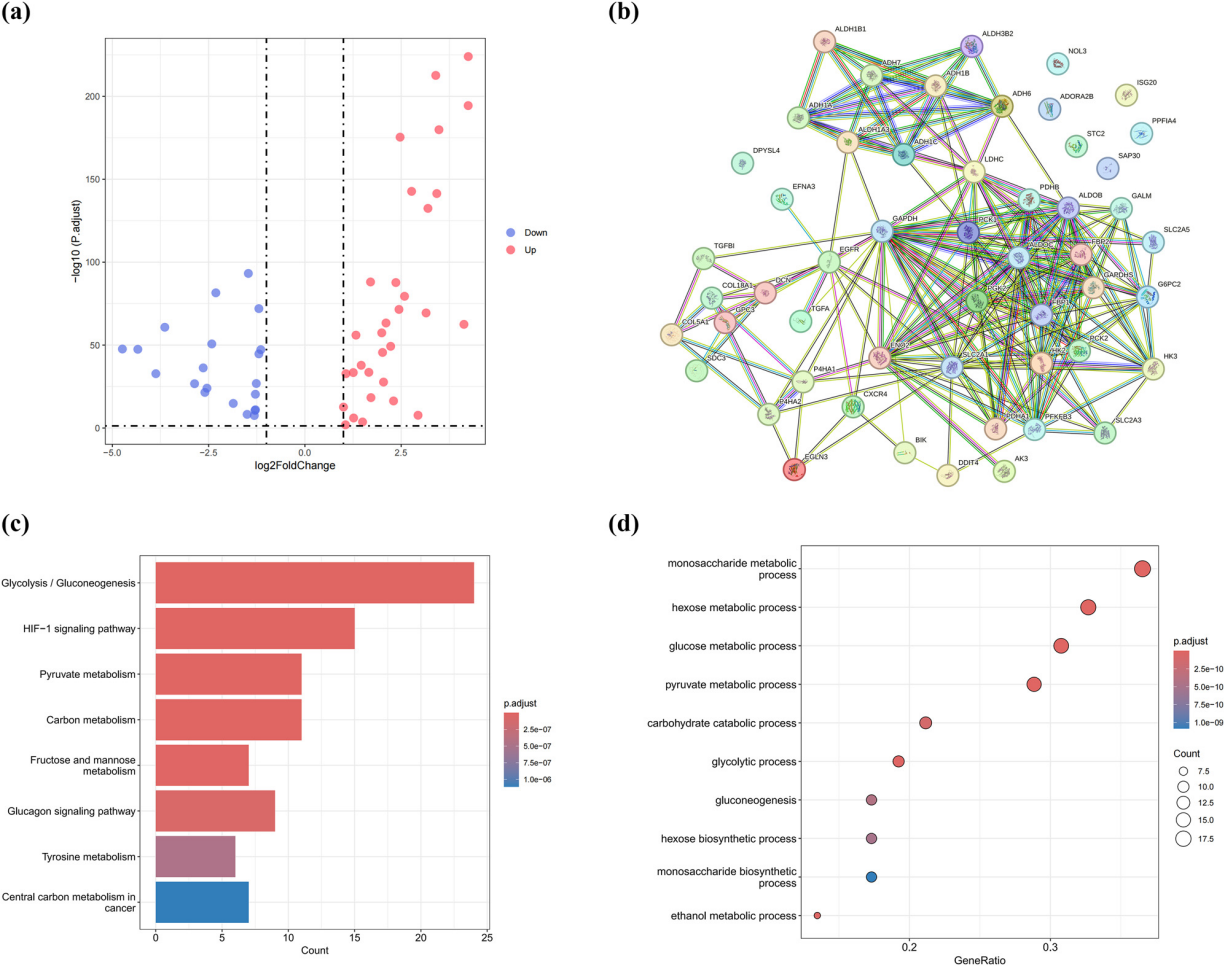
Screening for HGRGs. (a) Volcano plot of 52 differentially expressed HGRGs. (b) PPI network among 52 HGRGs. (c) and (d) KEGG and GO enrichment analysis of 52 HGRGs.

Figure 1 provides a schematic representation of the experimental framework through a flowchart visualization. From the TCGA-KIRC dataset, we identified 52 HGRGs using the DESeq2 package in R, comprising 31 upregulated and 21 downregulated genes (Figure 2a), which are listed in Table S2. Subsequently, STRING database’s online analytical tools were leveraged to develop a protein–protein interaction (PPI) network for 52 target HGRGs (Figure 2b). Additionally, 52 HGRGs underwent enrichment analyses through KEGG and GO (Figure 2c and d). Enrichment results showed that the highly enriched terms included glycolysis, HIF-1 signaling pathway, pyruvate metabolism, and carbon metabolism, suggesting the potential roles of HGRGs in tumor progression.

### 3.2 Machine learning-based establishment of prognostic signature

**Figure 3:**
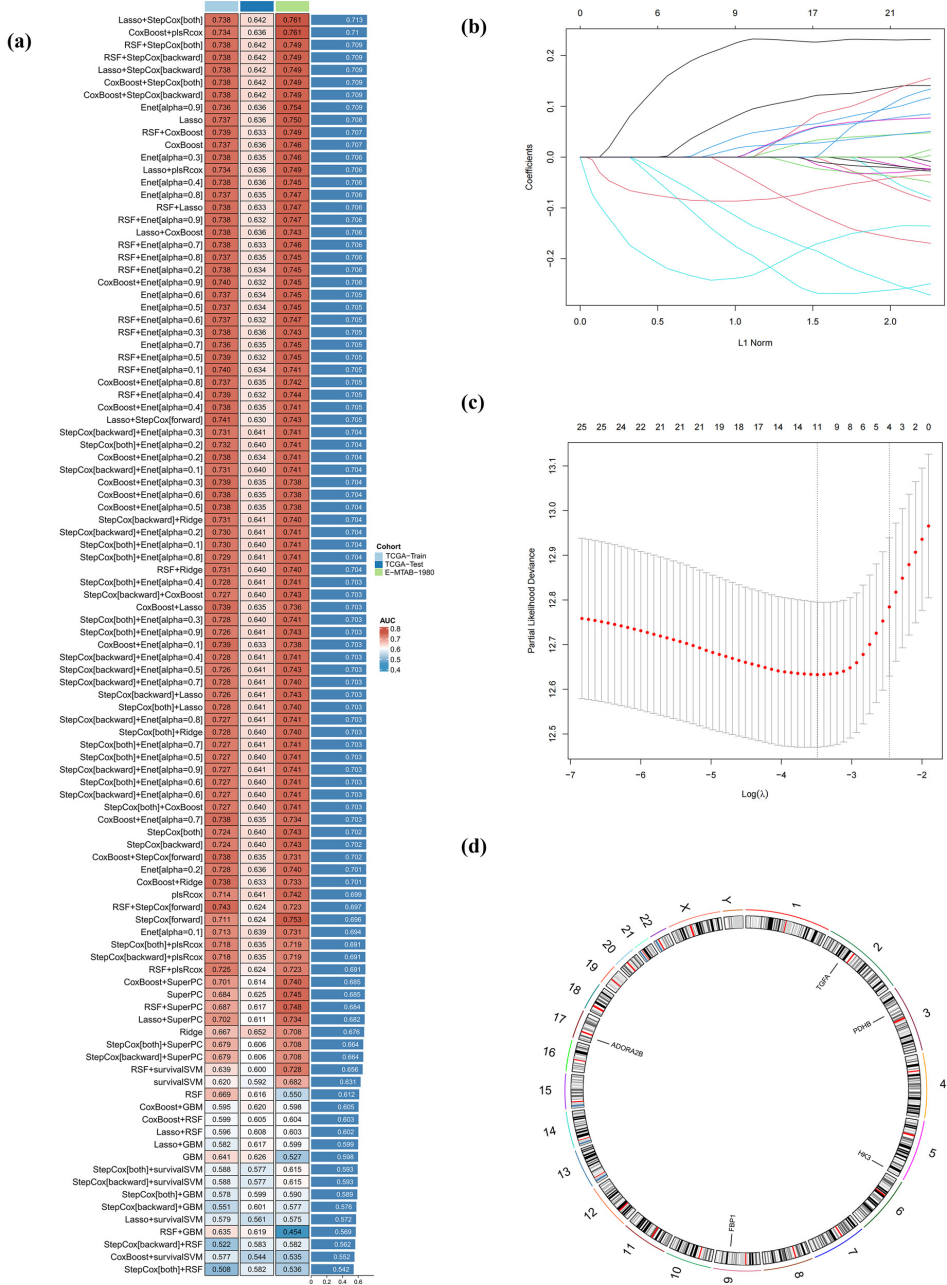
A prognostic model was constructed by machine learning. (a) C-index of each algorithm computed through the application of 101 algorithm combinations. (b) and (c) LASSO regression analysis. (d) Circle plot depicting chromosome locations of five model genes.

We randomized 529 TCGA-KIRC patients with the same sample size into training and testing groups, with E-MTAB-1980 utilized as an external testing group. Univariate Cox regression was implemented on the 52 HGRGs, and 25 genes were identified with prognostic values, which are shown in Table S3. We then employed 101 algorithm combinations to establish a prognostic hypoxia–glycolysis-related framework based on the expression of 25 HGRGs. 101 algorithm combinations within a cross-validation framework were implemented in the TCGA training cohort. Algorithm performance was quantified using Harrell’s C-index, with comparative results visualized in Figure 3a. We found that the integration of LASSO with Stepwise Cox (StepCox) yielded a maximum average C-index value of 0.713. Consequently, we applied these two algorithms to construct the prognostic model (Figure 3b and c). Ultimately, five HGRGs – adenosine A2b receptor (*ADORA2B*), transforming growth factor alpha (*TGFA*), fructose-1,6-bisphosphatase 1 (*FBP1*), hexokinase 3 (*HK3*), and pyruvate dehydrogenase E1 subunit beta (*PDHB*) – were selected for model construction (Table S4), with their chromosomal locations illustrated in Figure 3d. The correlation circle plot of five HRGs is shown in Figure S1a. The forest plots of five HGRGs associated with multifactor regression are illustrated in Figure S1b. Figure S1c indicates up- and down-regulation changes of five HGRGs.

### 3.3 Hypoxia–glycolysis-related prognostic model construction and validation

**Figure 4:**
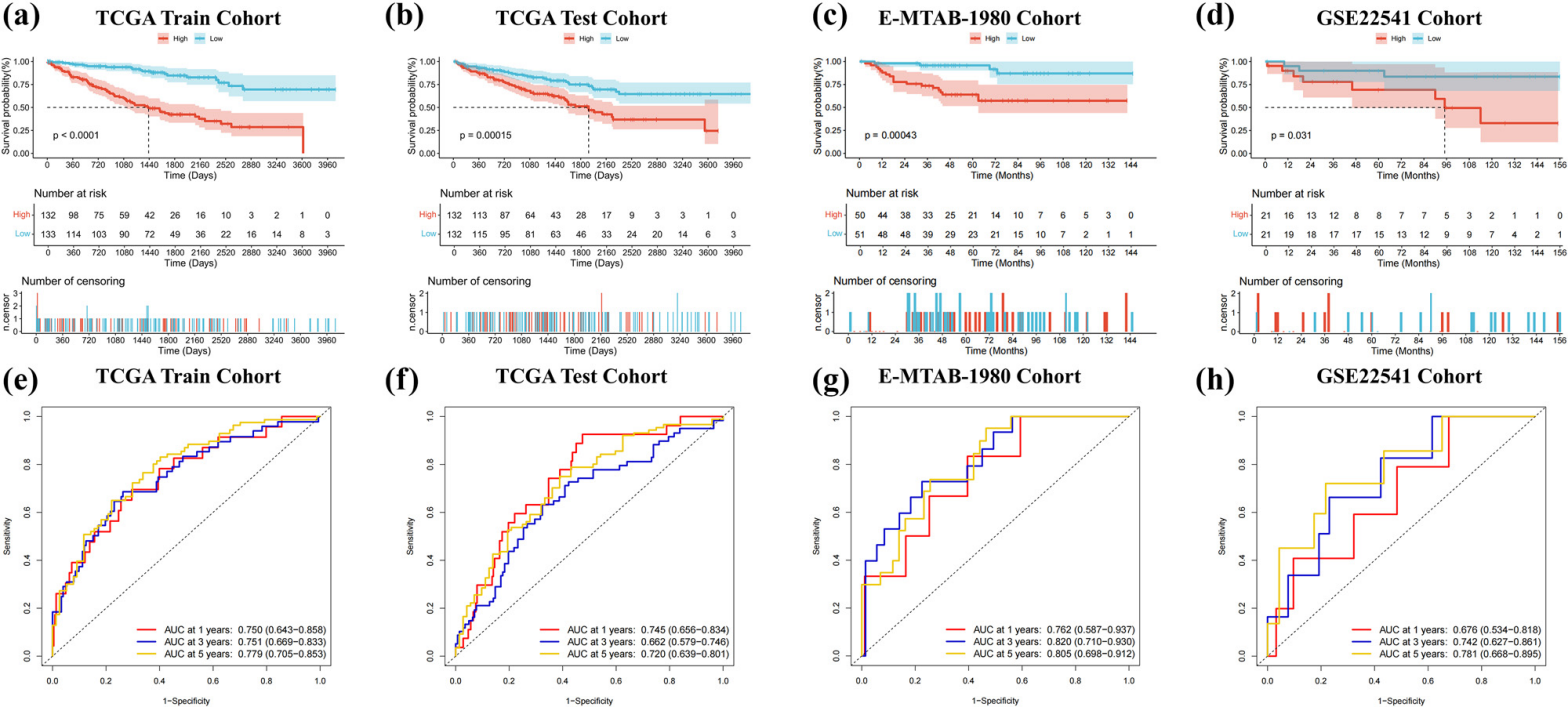
Model’s predictive capability is verified by the TCGA-KIRC, E-MTAB-1980, and GSE22541. (a)–(d) Survival outcomes between the HG and LG were compared through K–M curves. (e)–(h) ROC curves assessed the model’s predictive capability for survival outcomes among different groups.

**Figure 5:**
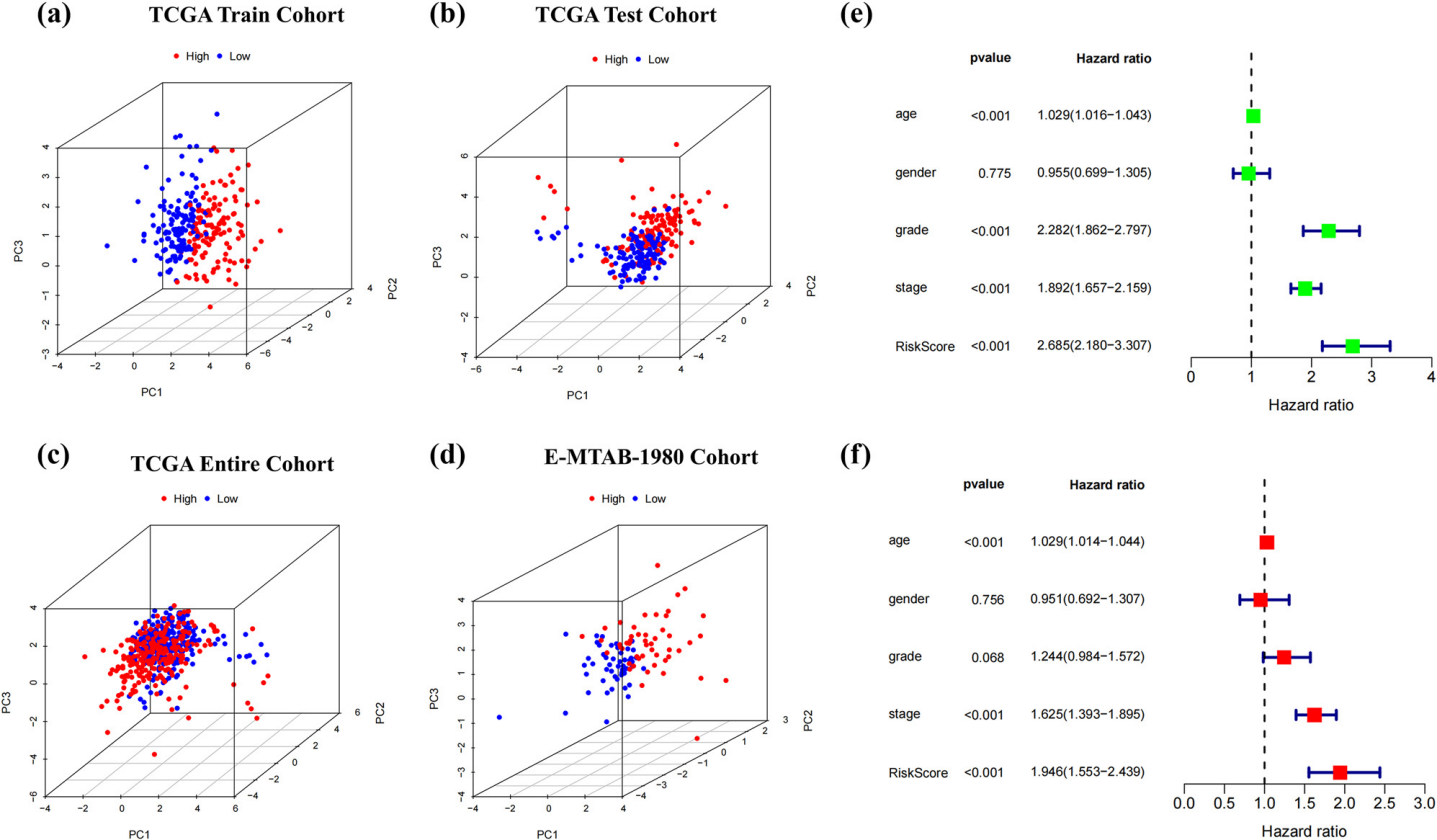
Validation of the predictive model was conducted using PCA and independent prognostic assessment. PCA results in (a) TCGA training cohort, (b) TCGA testing cohort, (c) TCGA entire cohort, and (d) E-MTAB-1980 cohort. (e) Univariate and (f) multivariate independent prognostic analysis.

Through the combination of LASSO with StepCox, we computed risk scores for all patients across TCGA-KIRC, E-MTAB-1980, and GSE22541 using the equation: Riskscore = 0.21640 × *ADORA2B* − 0.19359 × *TGFA* −0.24695 × *FBP1* + 0.40217 × *HK3* − 0.39773 × *PDHB*. Comprehensive clinical characteristics of patients stratified into risk categories are summarized in Table 2. K-M survival analysis performed on the training and testing groups of TCGA-KIRC, E-MTAB-1980, and GSE22541 demonstrated worse outcomes in the HG compared to the LG (Figure 4a–d). Additionally, the time-dependent ROC curves confirmed the model’s robust forecasting performance. The model demonstrated area under the curve (AUC) values of 0.750 (95% CI: 0.643–0.858), 0.751 (95% CI: 0.669–0.833), and 0.779 (95% CI: 0.705–0.853) at 1-, 3-, and 5-year intervals in the TCGA training cohort. Subsequent validation in the testing cohort yielded AUC scores of 0.745 (95% CI: 0.656–0.834), 0.662 (95% CI: 0.579–0.746), and 0.720 (95% CI: 0.639–0.801) for the corresponding time points. Further confirmation through the E-MTAB-1980 revealed predictive accuracy with AUC values reaching 0.762 (95% CI: 0.587–0.937), 0.820 (95% CI: 0.710–0.930), and 0.805 (95% CI: 0.698–0.912). In the GSE22541 cohort, the AUC values for 1-, 3-, and 5-year were 0.676 (95% CI: 0.534–0.818), 0.742 (95% CI: 0.627–0.861), and 0.781 (95% CI: 0.668–0.895) (Figure 4e–h). Furthermore, the heatmap showed the expression of the five HGRGs across different risk levels (Figure S2a–c), with expressions of ADORA2B and HK3 upregulated in the HG. The robust forecasting ability of the prognostic model was collectively demonstrated by risk curves, distribution plots, and scatter plots (Figure S2d–l). PCA results revealed distinct cluster separation between different risk subgroups across TCGA-KIRC and E-MTAB-1980 (Figure 5a–d). The univariate analysis conducted with TCGA-KIRC identified age (HR = 1.029, 95% CI: 1.016–1.043), grade (HR = 2.282, 95% CI: 1.862–2.797), stage (HR = 1.892, 95% CI: 1.657–2.159), and risk score (HR = 2.685, 95% CI: 2.180–3.307) as independent prognosis predictors (all *p*-value < 0.001). Subsequent multivariate Cox regression analysis further validated age (HR = 1.029, 95% CI: 1.014–1.044), stage (HR = 1.625, 95% CI: 1.393–1.895), and risk score (HR = 1.946, 95% CI: 1.553–2.439) as independent predictive indicators with persistent significance (all *p*-value < 0.001, Figure 5e and f).

**Table 2:** Clinical characteristics of 529 patients in the HG and LG

Characteristics	High-risk group (*n* = 264)		Low-risk group (*n* = 265)	
	*n*	Percentage	*n*	Percentage
**Age**				
<= 65	170	64.39	87	32.95
>65	94	35.61	178	67.42
**Status**				
Alive	135	51.14	221	83.71
Dead	129	48.86	44	16.67
**Gender**				
Female	77	29.17	108	40.91
Male	187	70.83	157	59.47
**Grade**				
G1	3	1.14	10	3.79
G2	85	32.20	143	54.17
G3	111	42.05	94	35.61
G4	61	23.11	14	5.30
Unknown	4	1.52	4	1.52
**Stage**				
Stage I	94	35.61	169	64.02
Stage II	29	10.98	28	10.61
Stage III	76	28.79	47	17.80
Stage IV	63	23.86	20	7.58
Unknown	2	0.76	1	0.38
**T stage**				
T1	98	37.12	171	64.77
T2	37	14.02	32	12.12
T3	118	44.70	62	23.48
T4	11	4.17	0	0.00
**M stage**				
M0	191	72.35	229	86.74
M1	59	22.35	20	7.58
Unknown	14	5.30	16	6.06
**N stage**				
N0	124	46.97	115	43.56
N1	12	4.55	4	1.52
Unknown	128	48.48	146	55.30

Abbreviations: T stage: tumor stage; N stage: node stage; M stage: metastasis stage.

**Figure 6:**
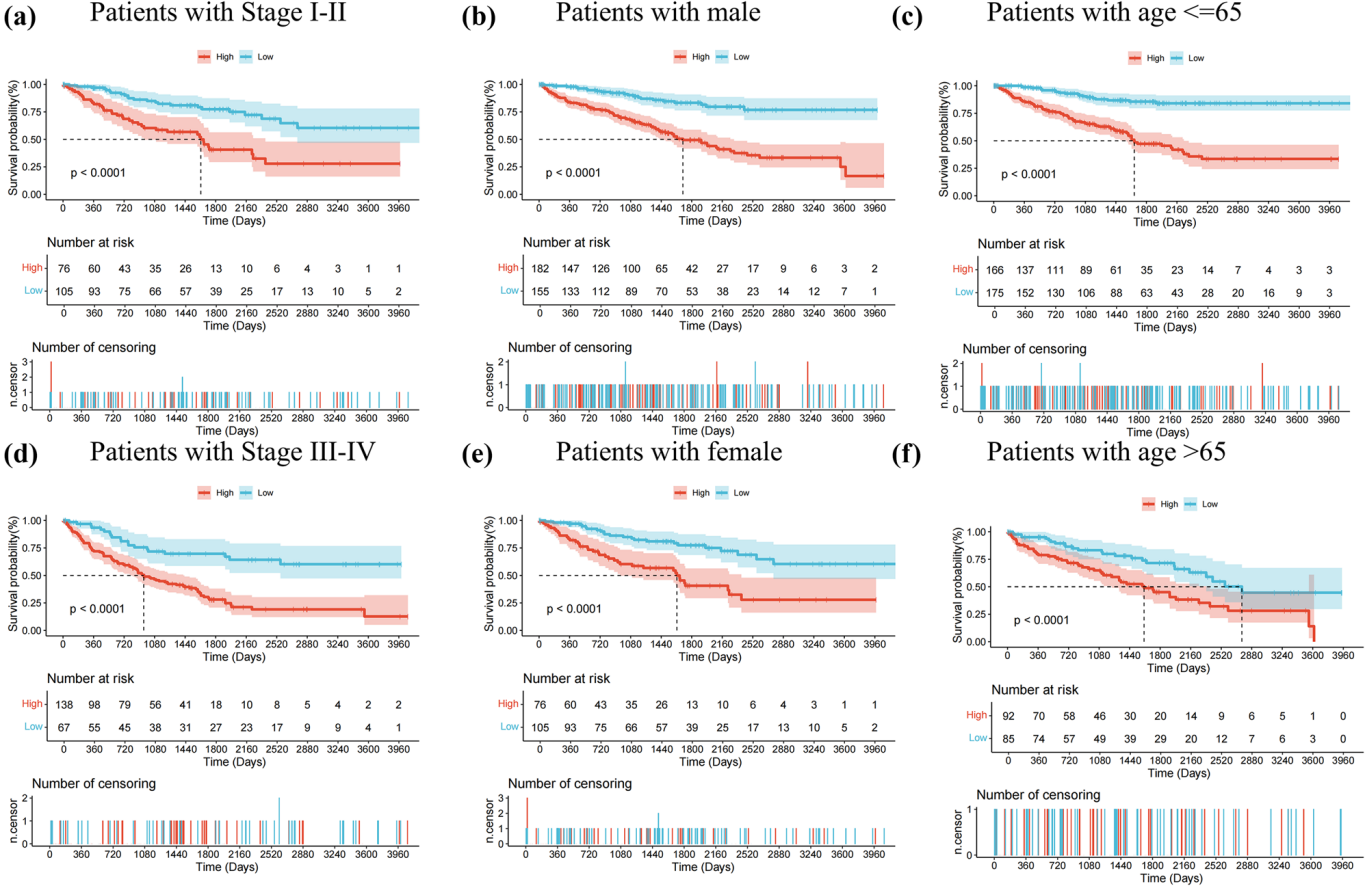
Additional subgroup verification demonstrating model efficacy. K–M survival analysis results conducted with stage, age, and gender (a)–(f).

The expression heatmap (Figure S3a), which integrates clinical information, risk subgroups, and five HGRGs genes, combined with K-M survival curves (Figure 6) validates the clinical relevance of the model’s predictions. Statistical analysis revealed notable differences in survival among the groups divided by clinical characteristics, including stage, gender, and age. Scatter plots combined with box plots demonstrated that no statistically significant correlation between risk scores generated by our prognostic model and age (*p* > 0.05). A statistically significant positive correlation was observed between tumor stage progression and elevated risk scores in the plot (*p* < 0.001) (Figure S3b and c).

### 3.4 Establishment of a clinical prediction nomogram

**Figure 7:**
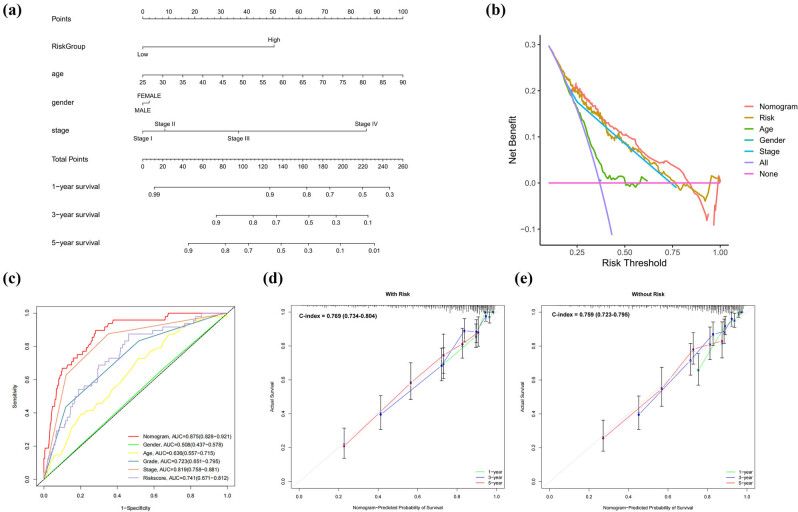
Patient prognosis is predicted through the nomogram model. (a) A prognostic nomogram incorporates risk, age, gender, and stage. (b) DCA evaluates the predictive utility. (c) ROC analysis integrates multiple clinical information. Comparative visualization demonstrates nomogram performance (d) with versus (e) without risk model.

Through incorporating the risk model, age, sex, and stage, we developed a prognostic nomogram for TCGA-KIRC patients (Figure 7a). DCA (Figure 7b) and ROC curves at 1-year (Figure 7c) revealed that the nomogram (AUC = 0.875, 95% CI: 0.828–0.921) and prognostic model (AUC = 0.741, 95% CI: 0.671–0.812) outperformed individual clinical factors, including gender (AUC = 0.508, 95% CI: 0.437–0.578), age (AUC = 0.636, 95% CI: 0.557–0.715), and grade (AUC = 0.723, 95% CI: 0.651–0.795). The forecasting capability of the nomogram was better than the stage (AUC = 0.819, 95% CI: 0.758–0.881, DeLong’s test: *p* = 0.010). The ROC curves of year-3 and year-5 are exhibited in Figure S4. Subsequent validation of this model demonstrated good performance (Figure S5). The integration of the risk model constructed by HGRGs yielded a numerically higher, but not statistically significant, C-index (0.769, 95% CI: 0.734–0.804) and showed a trend toward improved discriminative ability versus omitting it (C-index = 0.759, 95% CI: 0.723–0.795) (Figure 7d and e).

### 3.5 Enrichment analysis

KEGG enrichment analysis identified several functions associated with the five HGRGs, including glycolysis/gluconeogenesis, carbon metabolism, etc. (Figure S6a). GO enrichment analysis demonstrated cellular components and biological process functions such as carbohydrate phosphatase activity, fructose 6-phosphate metabolic process, etc. (Figure S6b) GSEA revealed that distinct biological pathways were significantly enriched in risk subgroups. The LG exhibited significant enrichment pathways including fatty acid metabolism, peroxisome, proximal tubule transport, lipid modification, etc. Furthermore, pathways that were significantly enriched in the HG included profibrotic mediator pathways, cytokine pathways, and DC, NKT, STEM pathways, etc. (Figure S7). Table S5 provides a comprehensive summary of significant pathways in different gene sets.

### 3.6 TMB

**Figure 8:**
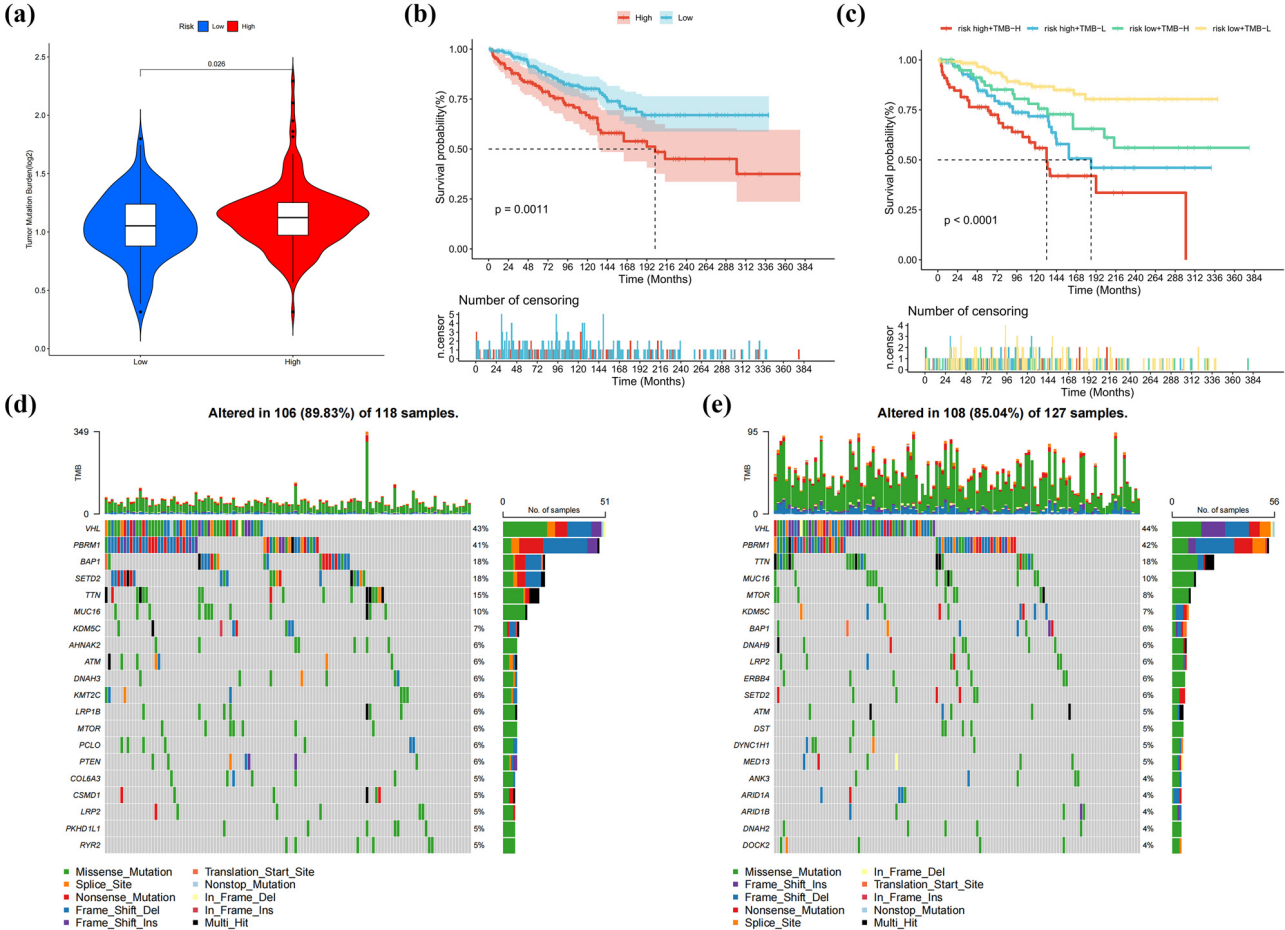
Mutational landscape characteristics in categorized risk groups. (a) Differential TMB between the HG and LG in KIRC. (b) K–M survival analysis for different TMB groups, (c) for a TMB combined with risk group. (d) Waterfall diagram for HG. (e) Corresponding visualization for LG.

Through analysis of TCGA somatic mutation datasets, the HG demonstrated increased TMB compared to the LG (Figure 8a). Survival analysis based on TMB levels revealed significantly worse clinical outcomes in the high-TMB cohort compared to the low-TMB cohort (Figure 8b). Moreover, individuals exhibiting elevated TMB combined with high-risk features experienced the worst adverse clinical outcomes (Figure 8c). The mutational landscape of the top 20 frequently altered genes, stratified according to risk group, was visualized through waterfall plots (Figure 8d and e). In HG patients, the five predominant genetic alteration genes were VHL (43%), PBRM1 (41%), BAP1 (18%), SETD2 (18%), and TTN (15%). Conversely, in LG patients, the five predominant genetic alterations were VHL (44%), followed by PBRM1 (42%), TTN (18%), MUC16 (10%), and MTOR (8%).

### 3.7 Tumor immune infiltration

**Figure 9:**
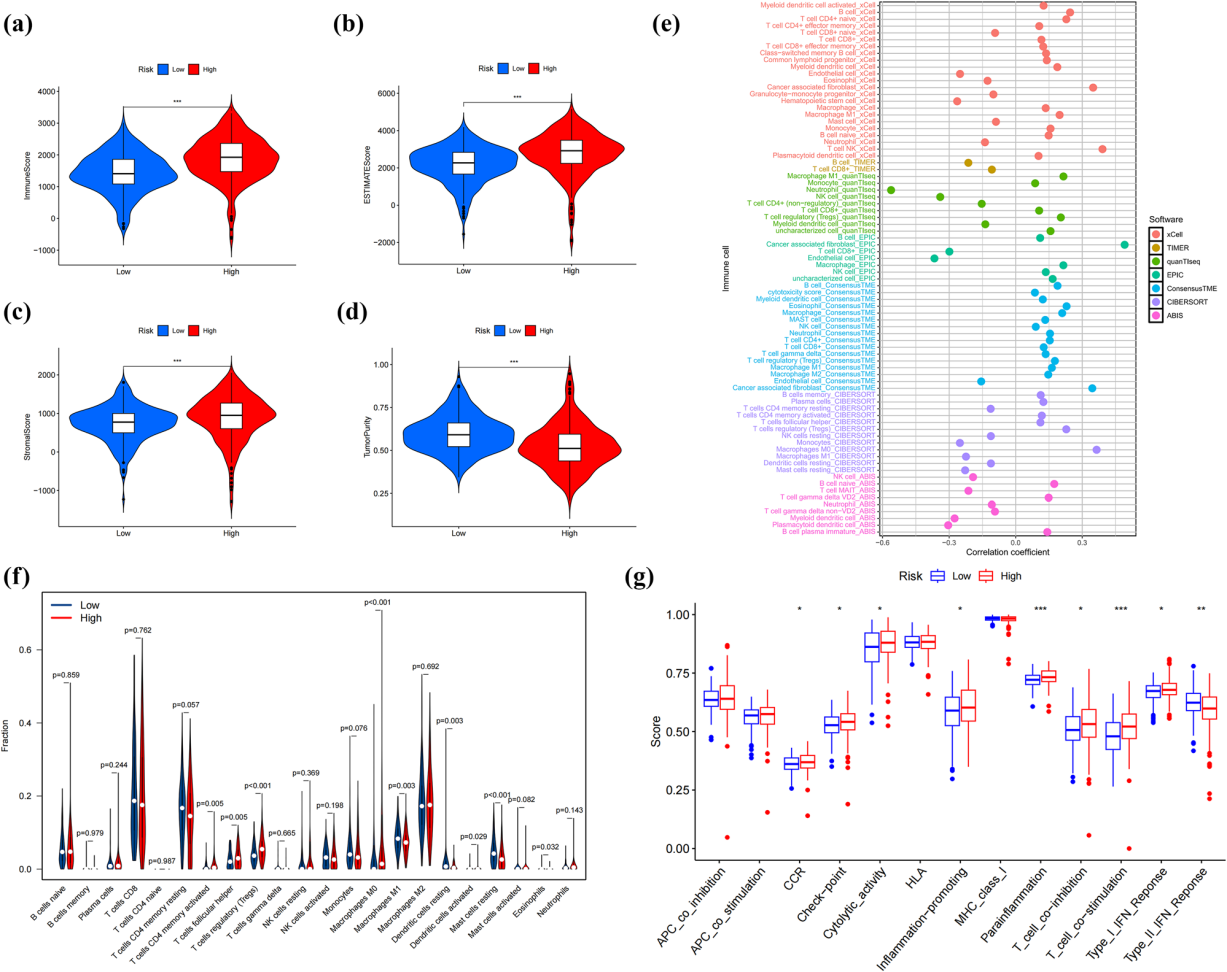
Comparatively investigation of the immune microenvironment between different prognostic categories. Violin plots of (a) immune scores, (b) ESTIMATE scores, (c) stromal scores, and (d) tumor purity across distinct prognostic categories. (e) Bubble plots of immune infiltration-risk association patterns using six methodologies. (f) CIBERSORT-based comparative analysis of 22 immune cells between prognostic groups. ssGSEA for comparing the immune functions between the HG and LG patients (g). *p < 0.05, **p < 0.01, ***p < 0.001.

TME analysis revealed significantly elevated immune scores (Figure 9a), stromal scores (Figure 9b), and ESTIMATE scores (Figure 9c) in HG patients compared to LG patients (*p* < 0.01). However, HG patients exhibited markedly lower tumor purity (*p* < 0.01) (Figure 9d). It was speculated that the HG represents a more immunosuppressed phenotype, which may weaken the immune response against cancer. To further explore the characteristics of the tumor immune microenvironment in the high and low groups, we used the CIBERSORT algorithm to calculate the estimated proportions of 22 types of immune cells in these groups (Figure S8a). A heatmap was used to visualize the immune features with statistically significant differences identified by six algorithms based on the IOBR package (Figure S8b). Furthermore, we identified associations between immune cell populations and risk scores across different algorithms (Figure 9e). Significantly increased proportions were detected in the HG regarding specific immune cells: M0-polarized macrophages, activated CD4 memory, follicular helper T cells, and T cells regulatory T cells (Tregs) (all *p*-value < 0.01). Conversely, the LG exhibited higher abundances of M1-polarized macrophages as well as resting-state mast cells (all *p*-values < 0.01) (Figure 9f). Figure S9 provides additional visualization of the correlations between immunological cell subtypes and risk scores. In the LG, the type II interferon response was significantly suppressed (*p* < 0.01). In the HG, inflammation promotion, functions related to chemokine receptor signaling, para-inflammation, type I interferon response, cytolytic activity, para-inflammation, immune checkpoint pathways, T-cell co-inhibition, and T-cell co-stimulation (all *p*-value < 0.05) were enhanced (Figure 9g).

### 3.8 Effects of prognostic model in immunotherapy and chemotherapy

Elevated T-cell dysfunction, along with TIDE levels, was observed in the HG (Figure S10). This finding suggests that tumors within the HG may possess an increased capacity for immune escape, complicating treatment strategies. The antitumor drugs to which HG tumors demonstrate sensitivity are listed in Table S6, while Table S7 outlines those that are effective for tumors in the LG. Table S8 lists those without significant susceptibility differences between the two groups. Notably, the HG demonstrates increased responsiveness to drugs targeting DNA replication pathways such as Camptothecin and Cisplatin, whereas the LG shows heightened sensitivity to agents that act on epidermal growth factor receptor (EGFR) signaling such as Gefitinib and Afatinib. These insights can inform the tailored application of antitumor therapies for patients categorized into different risk groups.

### 3.9 Immunohistochemistry staining and RT-qPCR

**Figure 10:**
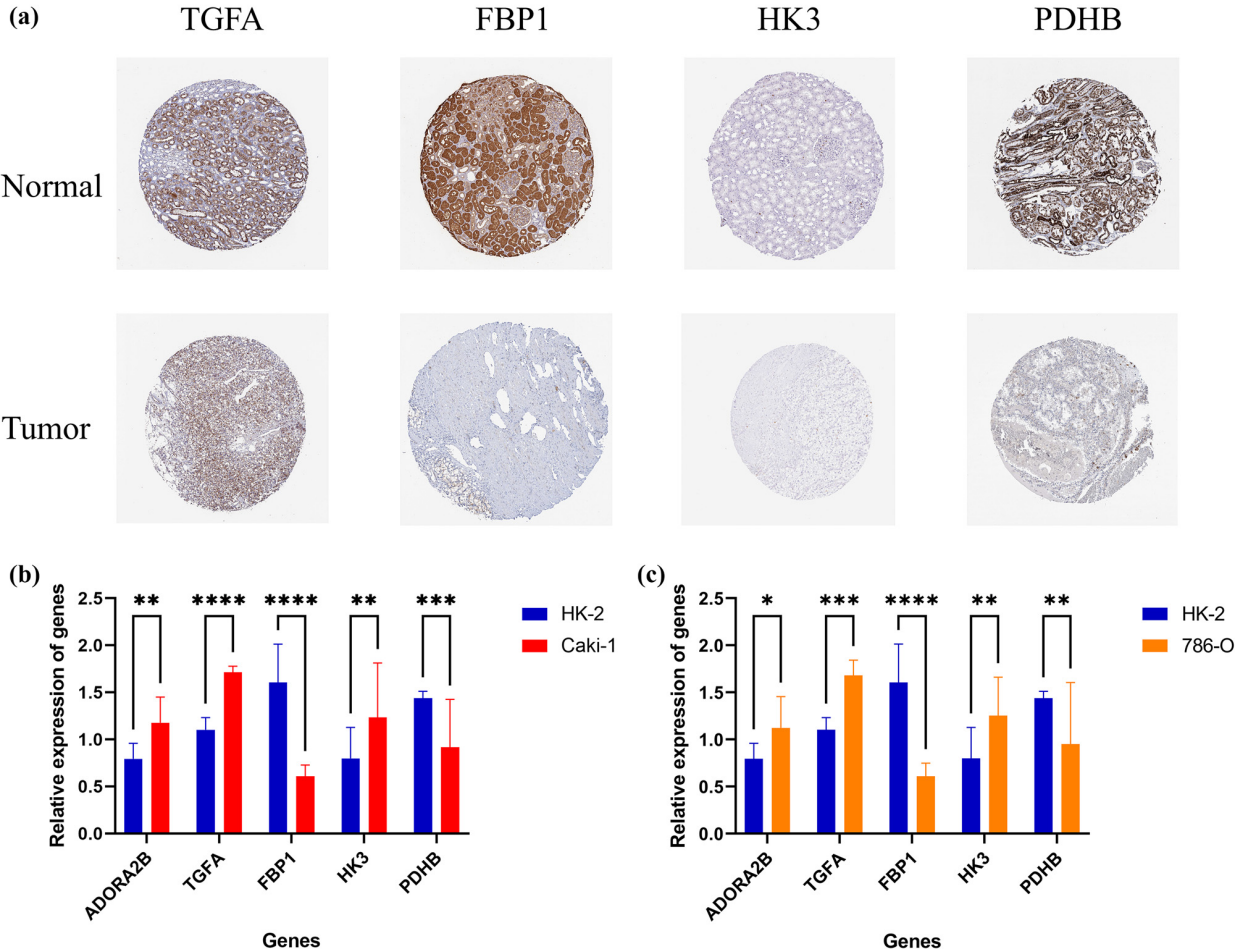
Immunohistochemistry staining and *in vitro* assays of HGRGs. (a) Comparative immunohistochemical profiles of selected HGRGs in tumor or normal tissues. Relative expressions of five HGRGs in HK-2, (b) CAKI-1, and (c) 786-O cell lines. *p < 0.05, **p < 0.01, ***p < 0.001, ****p < 0.0001.

In the HPA database, PDHB and FBP1 proteins exhibited comparatively diminished abundance levels in KIRC. However, TGFA and HK3 were relatively highly expressed (Figure 10a). Relative expressions of the five HGRGs in HK-2, CAKI-1, and 786-O were quantified by RT-qPCR analysis (Figure 10b and c). PDHB and FBP1 protein expressions were lower compared with HK-2, whereas the expression levels of ADORA2B, TGFA, and HK3 were relatively high. These results showed remarkable consistency with our former analysis of the sequencing data.

## 4 Discussion

KIRC represents nearly four-fifths of all RCC cases observed in adult populations [22] and is characterized by its high propensity for metastasis. Hypoxia and glycolysis, hallmarks of solid tumors [23,24], serve as key factors in modulating the tumor immune microenvironment and promoting accelerated oncogenesis [25,26]. We identified HGRGs, precipitating the formulation of a KIRC prognostic framework coupled with underlying molecular mechanisms and potential applications. Our findings indicate that patients in the HG exhibit poorer clinical outcomes. The nomogram, based on the prognostic model and clinical data demonstrates good predictive value. Additionally, inflammatory responses were significantly enriched in HG. Immunosuppression and elevated TIDE scores were demonstrated in the HG patients, suggesting a heightened resistance to immunotherapy.

Our research developed a new predictive model through analysis of five HGRGs, which demonstrated high accuracy in predicting patient outcomes. We identified 25 HGRGs and created a prognostic framework based on 101 algorithm combinations. Among the various algorithm combinations evaluated, the LASSO and StepCox [both] combinations yielded the highest C-index. Consequently, we adopted this approach to construct the prognostic model, ultimately selecting five key genes for model development: ADORA2B, TGFA, FBP1, HK3, and PDHB. Patients categorized in the HG demonstrated markedly lower survival rates when contrasted with the LG. The model demonstrated robust predictive validity across the training, internal, and external validation cohorts. Through incorporating clinical traits with risk scores, we established a nomogram to forecast the prognosis for KIRC patients. Integrating the risk score enhanced the model’s forecasting performance compared with versions without it. The nomogram based on the prognostic model demonstrates a greater prognostic value compared to the traditional TNM staging system.

Apoptosis is a key mechanism in tumor progression, and there is a close correlation between the hypoxic–glycolytic microenvironment and apoptosis resistance [27]. The poor prognosis of high-risk patients may be related to escaping apoptosis. ADORA2B is a gene encoding the adenosine A2B receptor, a member of the adenosine G protein-coupled receptor family that is involved in regulating inflammation, hypoxia responses, and tumor progression. It has been reported that ADORA2B can promote the proliferation of various cancer cell types and confer apoptosis resistance by activating the PI3K–Akt signaling pathway [28]. FBP1, a key enzyme in gluconeogenesis, is downregulated in RCC. FBP1 functions as a protein phosphatase and can inhibit PPARα-driven β-oxidation gene expression by dephosphorylating histone H3 at threonine 11, thereby promoting apoptosis under conditions of energy stress [29]. The TNF-α signaling pathway plays an important role in tumor progression. It mediates cancer-related inflammation, regulates cytokines, enhances tumor invasiveness, and accelerates tumor progression through activation of the NF-κB and MAPK pathways. Hypoxia and glycolysis are closely associated with the TNF-α signaling pathway. Relevant studies have shown that under hypoxic conditions, tumor hypoxic regions produce large amounts of pro-inflammatory mediators, which activate NF-κB and enhance the inflammatory and anti-apoptotic capacities of tumor cells [30]. TNF-α enhances glycolysis and lactate production in tumor cells via the NF-κB signaling pathway, promoting the expression of GLUT1 and hexokinase 2 (HK2), and thereby facilitating tumor progression [31]. PDHB is a key subunit of the pyruvate dehydrogenase complex, and its function is regulated by TNF-α. TNF-α can modulate PDH metabolic flux and alter the expression of downstream inflammatory genes [32]. These results suggest that the TNF-α signaling pathway may be closely related to the function of HGRGs, thereby affecting the prognosis of KIRC.

GSEA demonstrated notable enrichment in biological pathways in the LG, including fatty acid metabolism and peroxisomal functions. Fatty acid metabolism is a defining trait of RCC and represents a critical adaptive mechanism for tumor survival, demonstrating its viability as a drug target in RCC [33]. Specifically, malonyl-CoA decarboxylase, which is involved in fatty acid catabolism, has been shown to disrupt lipid homeostasis and thereby inhibit the progression of RCC [34]. In the HG, the proinflammatory pathway and profibrotic mediator pathway were enriched. As a key mediator driving renal fibrosis, TGF-β demonstrates a strong correlation with the epithelial–mesenchymal transition (EMT) phenomenon occurring in proximal tubule cells during nephropathy progression [35]. EMT is activated in tumor cells and mediates the invasive behavior and metastatic dissemination of malignant tumors [36]. The K-M survival curve based on the TMB group indicated that an elevated TMB is correlated with a poorer clinical outcome in patients. Elevated recruitment of immunosuppressive cell populations, including Tregs and M0 macrophages, was observed in HG patients. Comparative analysis demonstrated a marked reduction of resting-state mast cell populations and dendritic cell infiltration when contrasting HG samples with LG samples. Collectively, these findings indicate that the HG may exhibit a more immunosuppressive phenotype. Furthermore, patients classified in the HG displayed heightened stromal and immune activity, which may portend an increased risk for disease progression, potentially attributed to a more aggressive TME [37]. TIDE emerges as a pivotal determinant during neoplastic evolution and serves as a pivotal predictor of immunotherapy efficacy in oncology. In HG patients, we observed elevated TIDE scores alongside immune dysfunction, reinforcing the hypothesis that immune evasion correlates with HG patients.

Recent advances have shown that the application of multi-omics technologies has become a research hotspot in drug discovery, particularly in the identification of therapeutic targets for cancer [38,39]. Given the heterogeneity of KIRC tumors and the diverse responses to various therapeutic agents, we undertook a drug sensitivity analysis. The LG exhibited elevated sensitivity to EGFR targeting drugs, such as Erlotinib, Afatinib, and Gefitinib. In contrast, the HG demonstrated greater responsiveness to DNA replication inhibitors, including Camptothecin, Cisplatin, and Irinotecan. Collectively, the HGRGs prognostic signature provides valuable clinical insights for the selection of immunotherapy and chemotherapy regimens.

This study stands out for its several significant contributions to the hypoxia–glycolysis-related signatures in predicting outcomes for KIRC patients. Notably, it is the first study to integrate 101 algorithm combinations alongside HGRGs to develop a prognostic model for KIRC. Moreover, comprehensive analyses were conducted to compare the existing research in this field to assess its reliability with our model. First, in comparison to the hypoxia-related model by Ning et al. [8], which reported a 1-year AUC of 0.711 and a 3-year AUC of 0.708, our prognostic model is superior in forecasting the clinical outcomes at 1 and 3 years. Second, in comparison with the GRGs model established by Xing et al. [9], we validated our model internally and externally in a more comprehensive way. Third, compared with the prognostic model based on hypoxia and glycolysis constructed by Mao et al., we conducted an investigation to explore candidate therapeutic agents in patient cohorts categorized by differential risk groups [40]. In clinical practice, our prognostic model can be integrated into hospital electronic health record systems to automatically calculate patient risk scores in real time. At the initial diagnosis stage, the model can assist clinicians in risk stratification and provide personalized treatment plans, such as selecting more aggressive therapies for high-risk patients or opting for conservative management for low-risk patients. During treatment and subsequent follow-up, if continuous sample collection is possible, dynamic monitoring of prognostic risk scores can guide timely adjustments to therapeutic strategies.

Nevertheless, the limitations inherent in this research must be acknowledged. First, although our model has demonstrated consistency and excellent performance across three independent sample cohorts (TCGA-KIRC, E-MTAB-1980, and GSE22541), a significant limitation is the absence of Asian populations in the samples. Racial and geographic differences may potentially influence gene expression and model reliability, necessitating further validation of the model’s reliability in Asian populations in future studies. Second, the data used to construct our prognostic model originated from public databases such as TCGA and GEO. Samples inevitably face various influences, including patient inclusion criteria, data completeness, or potential selection bias toward larger tumor volumes or specific subtypes during sequencing. This may not fully represent the spectrum of KIRC cases encountered in routine clinical practice. When applied to complex real-world clinical samples, the model’s predictive performance may be compromised. Therefore, its efficacy requires further validation through prospective, multicenter clinical trials in independent cohorts. Finally, applying the model to clinical practice presents numerous challenges. For instance, while this study’s prognostic model is based on RNA-seq data, this technology remains difficult to implement as a routine diagnostic tool in clinical settings. Additionally, variations in processing workflows across different data sources for clinical samples could significantly impact model reproducibility. Furthermore, clinical implementation must consider economic feasibility and tangible improvements in clinical decision-making. Addressing these aspects requires exploring appropriate solutions in subsequent research.

## 5 Conclusion

In conclusion, we identified five HGRGs and constructed a prognostic model, demonstrating strong predictive value and clinical applicability. In addition, our findings revealed that profibrotic mediators and high TMB might be instrumental in high-risk patients. This investigation provides critical insights into therapeutic methods in KIRC management. However, given various limitations, additional experimental investigations are imperatively needed to explore the fundamental mechanisms, and our model requires validation in broader clinical trials.

## Abbreviations

ADORA2B: adenosine A2b receptor
AUC: area under the curve
DCA: decision curve analysis
DEGs: differentially expressed genes
EGFR: epidermal growth factor receptor
FBP1: fructose-1,6-bisphosphatase 1
FDR: false discovery rate
GSEA: gene set enrichment analysis
GO: gene ontology
GRGs: glycolysis-related genes
HG: high-risk group
HGRGs: hypoxia–glycolysis-related genes
HK3: hexokinase 3
HPA: Human Protein Atlas
HRGs: hypoxia-related genes
IC50: half maximal inhibitory concentration
KEGG: Kyoto encyclopedia of genes and genomes
K-M: Kaplan–Meier
KIRC: kidney renal clear cell carcinoma
MsigDB: Molecular Signatures Database
LG: low-risk group
PCA: principal component analysis
PDHB: pyruvate dehydrogenase E1 subunit beta
PPI: protein–protein interaction
RCC: renal cell carcinoma
ROC: receiver operating characteristic
StepCox: stepwise Cox
TCGA: The Cancer Genome Atlas
TGFA: transforming growth factor alpha
TIDE: tumor immune dysfunction and exclusion
TMB: tumor mutational burden
TME: tumor microenvironment
TNM: tumor–node–metastasis
TPM: transcripts per million

## Supplementary Material

Supplementary material
