# Supplementary material for: Identification and external validation of a prognostic signature based on hypoxia–glycolysis-related genes for kidney renal clear cell carcinoma
Source: Open Med (Wars). 2025 Oct 31;20(1):20251305. doi: 10.1515/med-2025-1305 (PMC12596871; doi:10.1515/med-2025-1305)
Supplement: Supplementary material [file med-2025-1305-sm.pdf]

Supplementary material

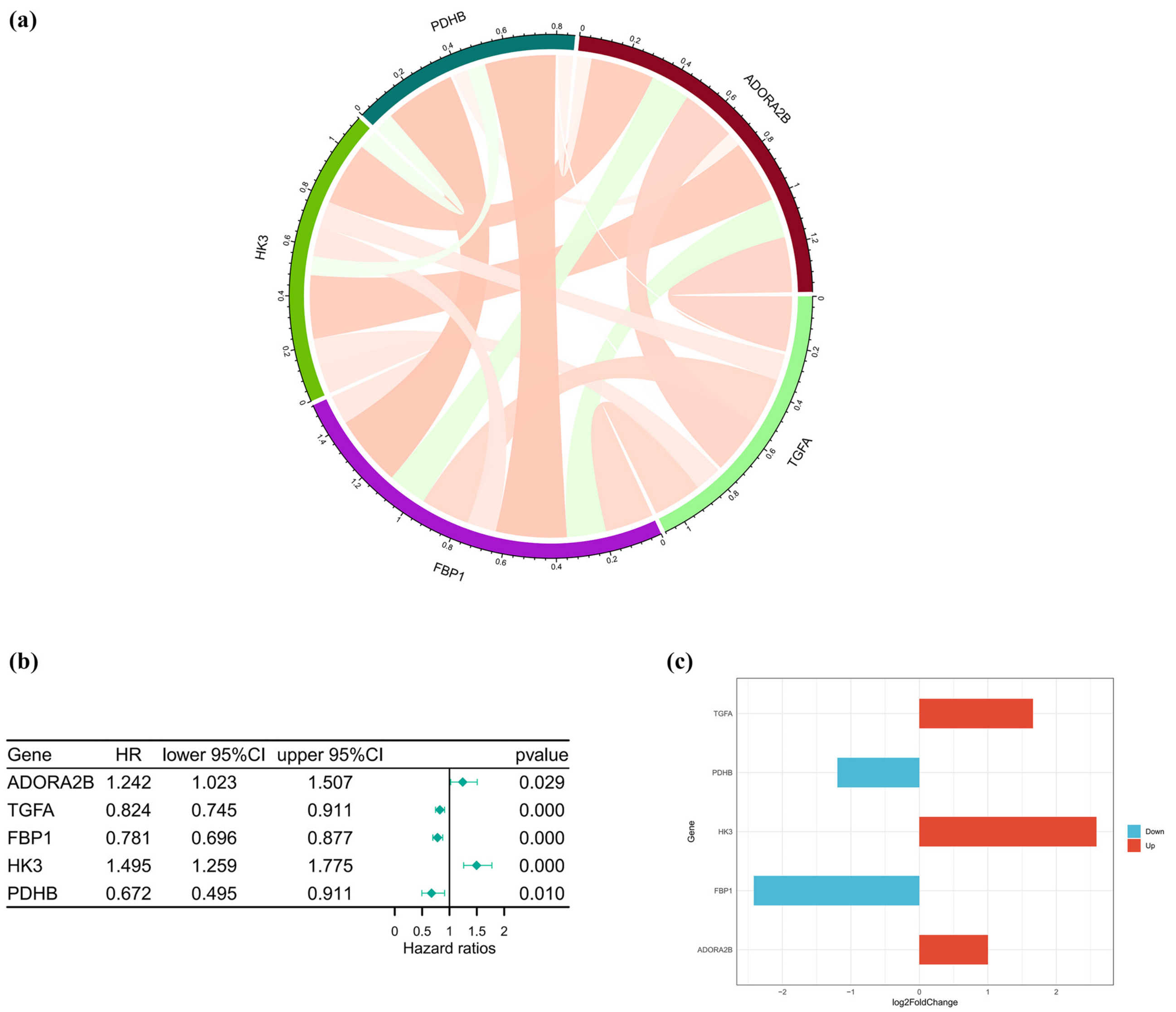

Figure S1: Construct a prognostic model. (a) Correlation circle plot of five HGRGs; (b) Forest plots associated with stepwise regression; (c) up- and down-regulation alterations in five HGRGs are illustrated in the deviation plots.

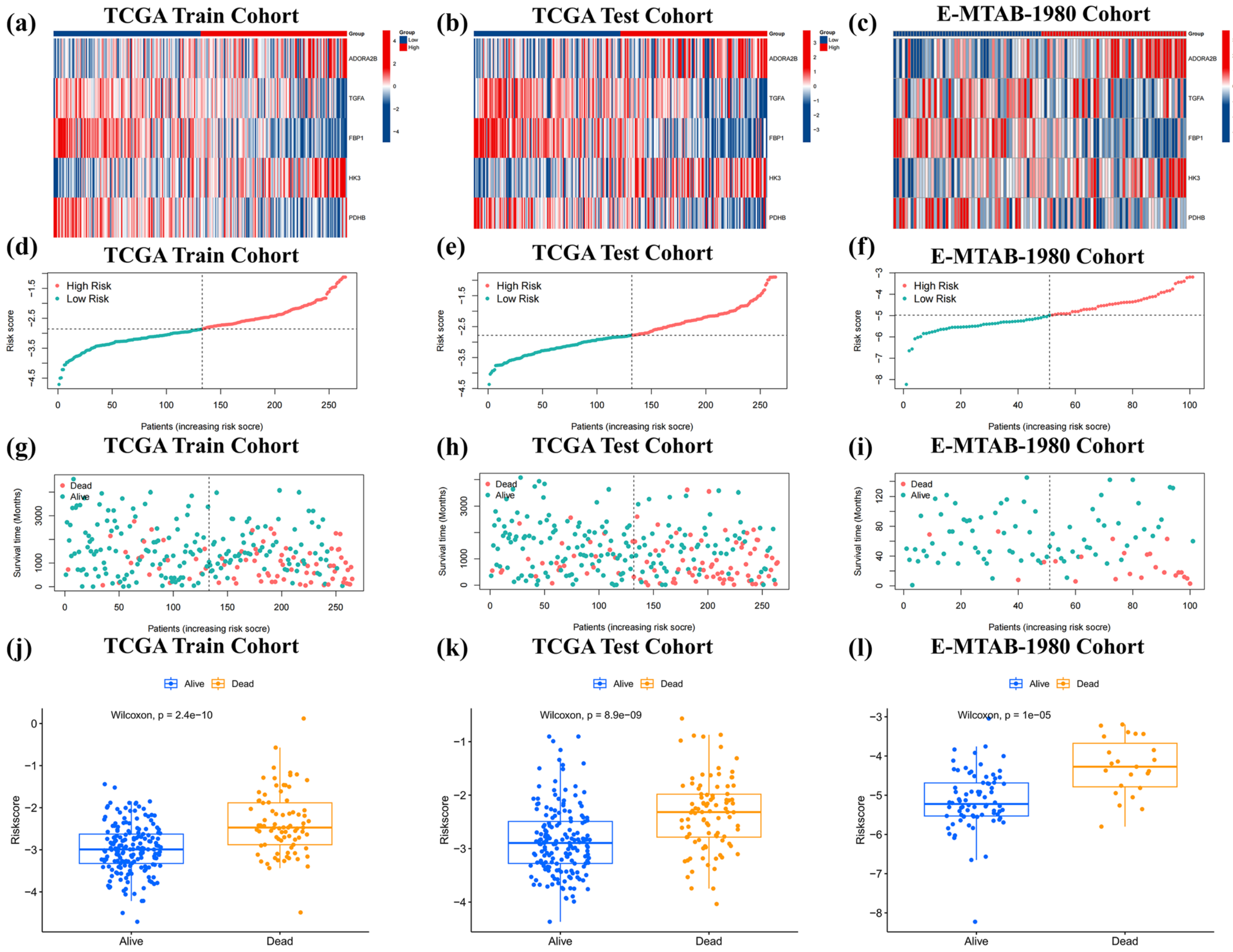

Figure S2: TCGA-KIRC and E-MTAB-1980 validated the model's forecasting capability. (a)–(c) Expression heatmaps of five hypoxia-glycolysis-related genes. (d)–(f) Prognostic risk curves demonstrating score distribution accompanied by (g)–(i) survival status scatterplots. (j)–(l) Visualization of risk score variations among patients stratified by survival outcomes.

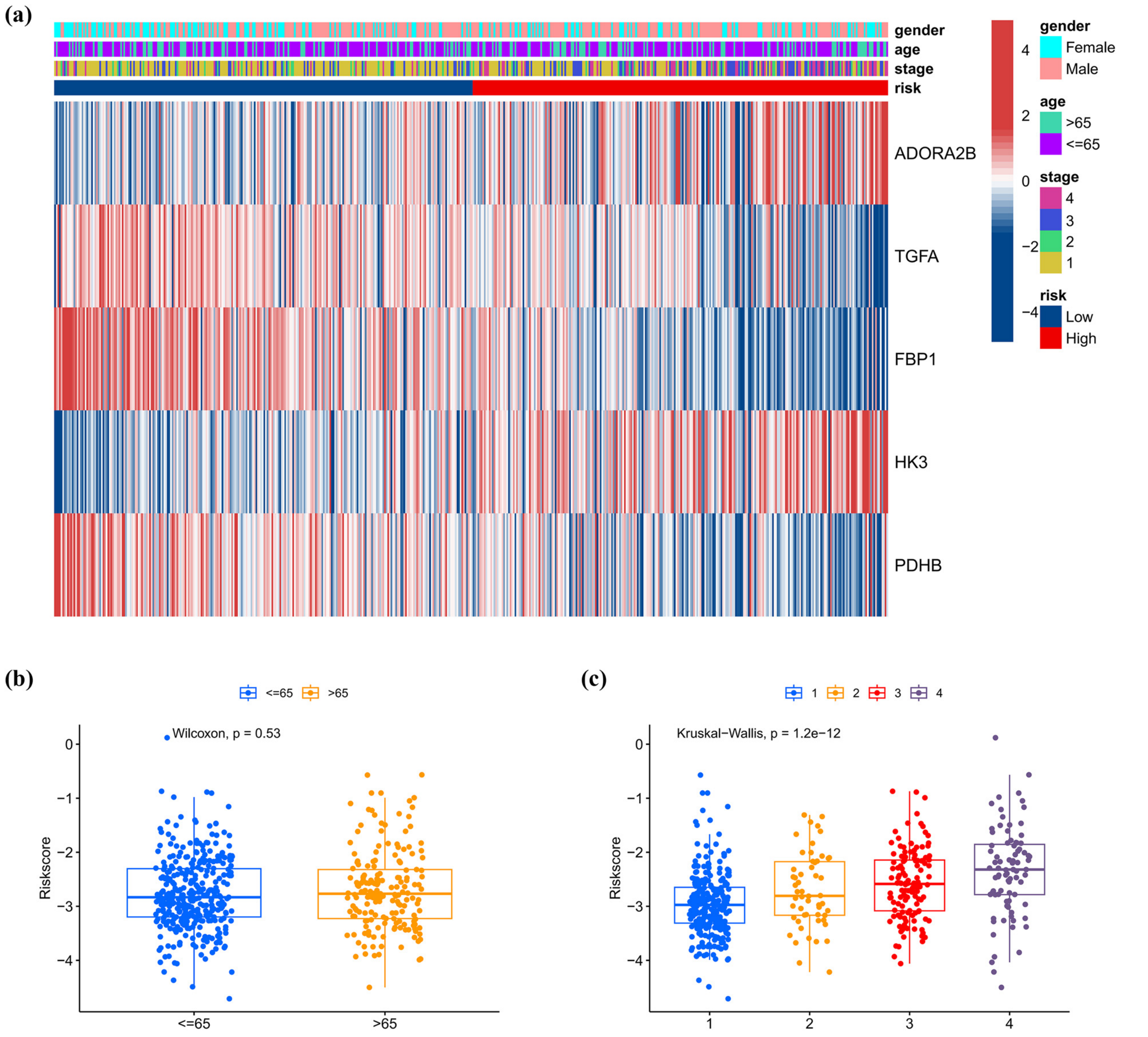

Figure S3: Additional verification of model effects. (a) Clinical information-integrated heatmap of five HGRG expressions; (b) and (c) Age-stratified and stage-dependent associations with risk score.

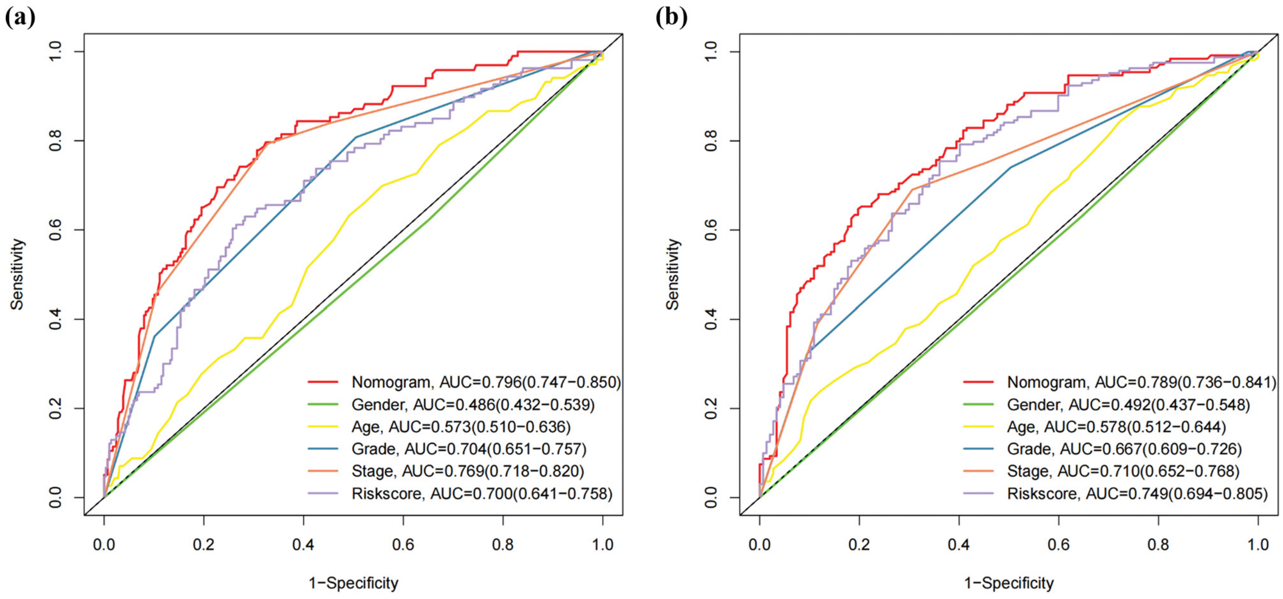

Figure S4: ROC analysis integrates nomogram and multiple clinical information at (a) year 3 and (b) year 5.

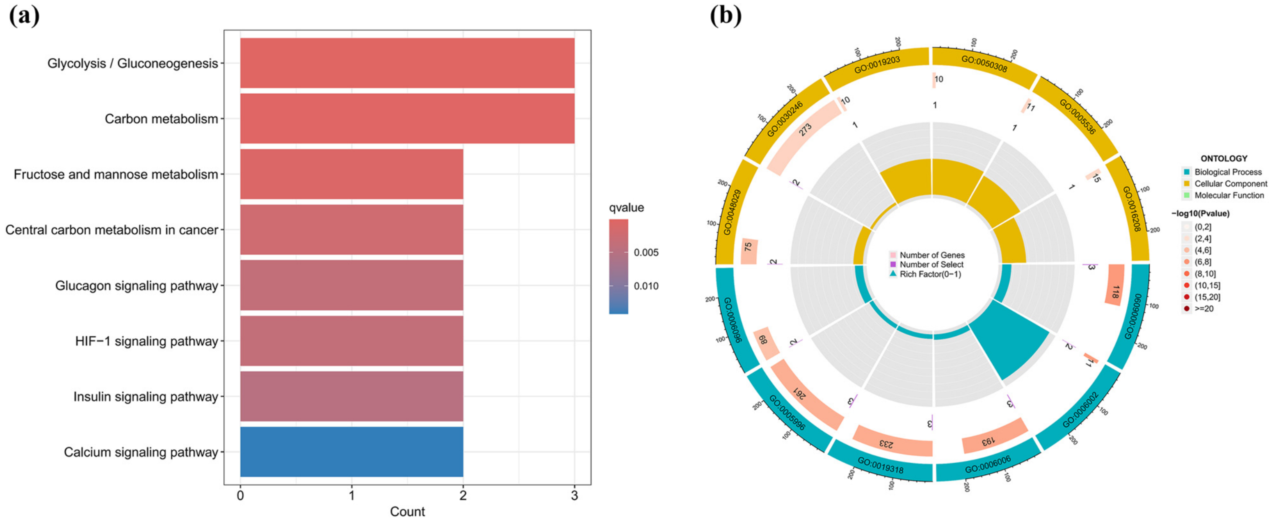

Figure S5: Nomogram with clinical characteristics.

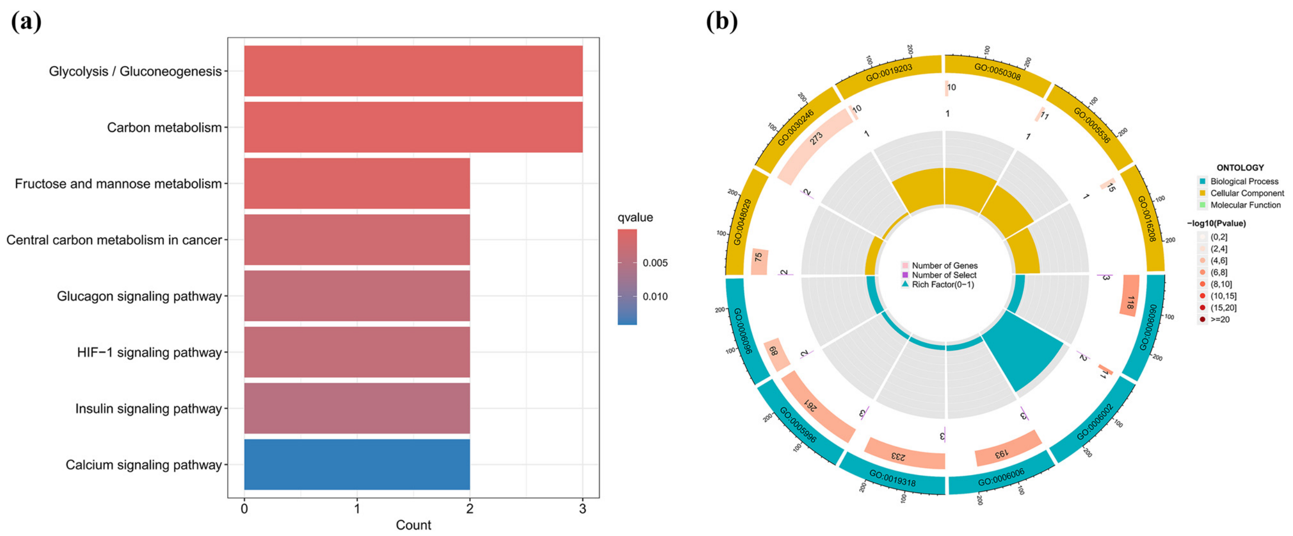

Figure S6: Enrichment analysis of five HGRGs. (a) KEGG; (b) GO.

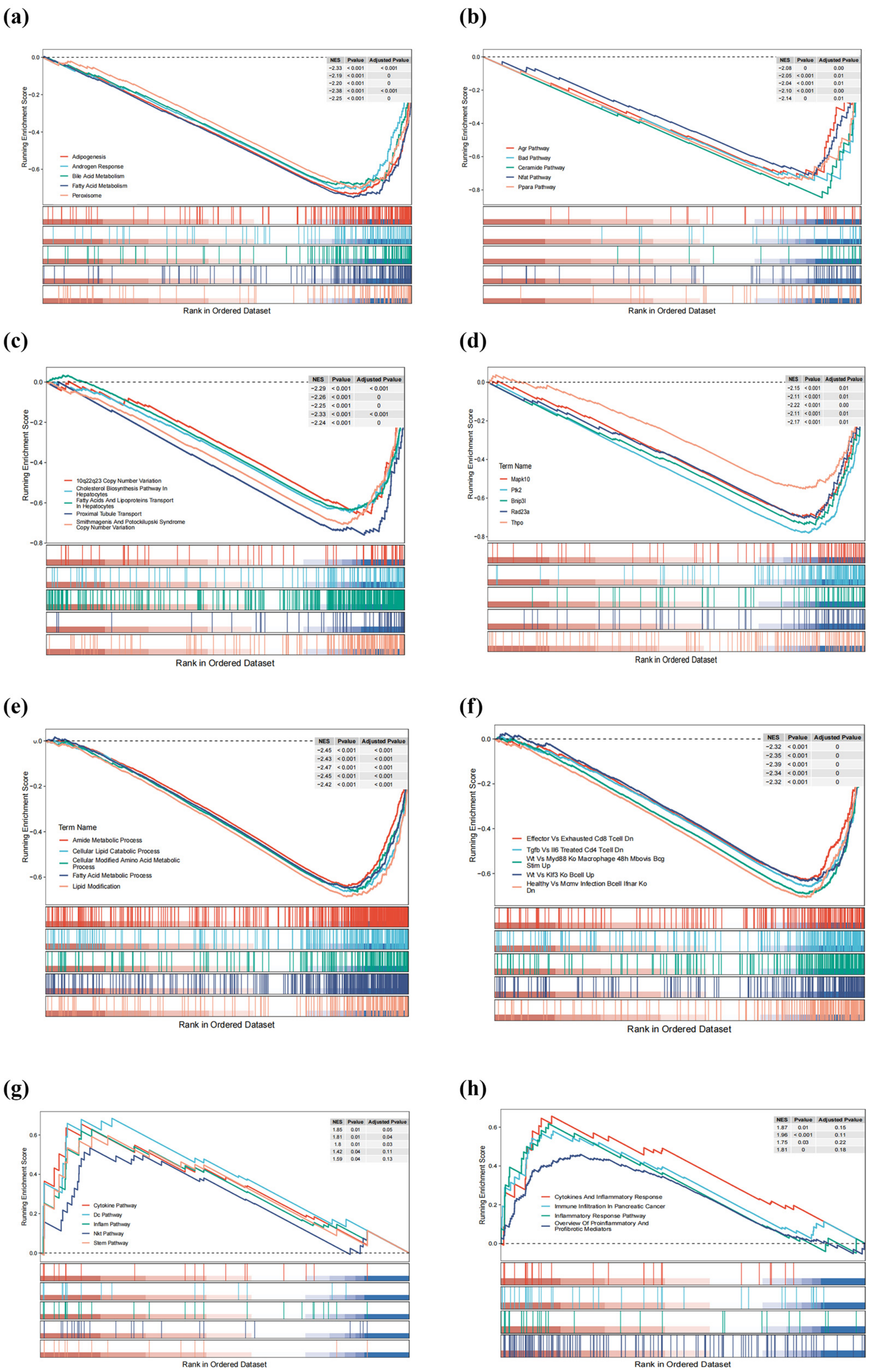

Figure S7: GSEA results between the LG and the HG. (a)–(f) Enrichment pathways enriched in the LG; (g) and (h) enrichment pathways enriched in the HG.

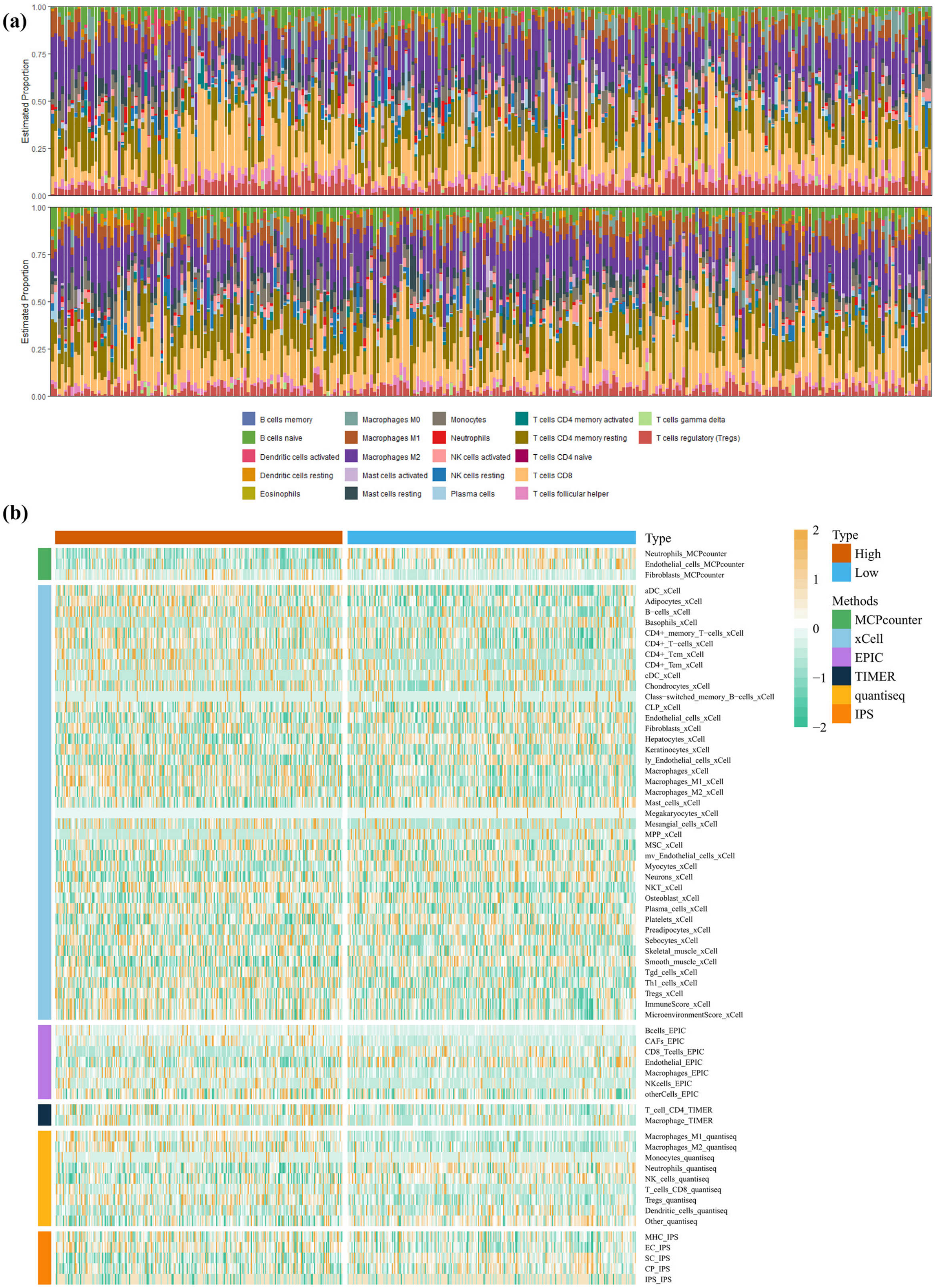

Figure S8: Comprehensive and systematic analysis with the IOBR package. (a) The abundance of 22 different immune cells between the high- and low-risk subgroups based on the CIBERSORT algorithm. (b) Immune cell infiltration was calculated by six algorithms between the high and low groups.

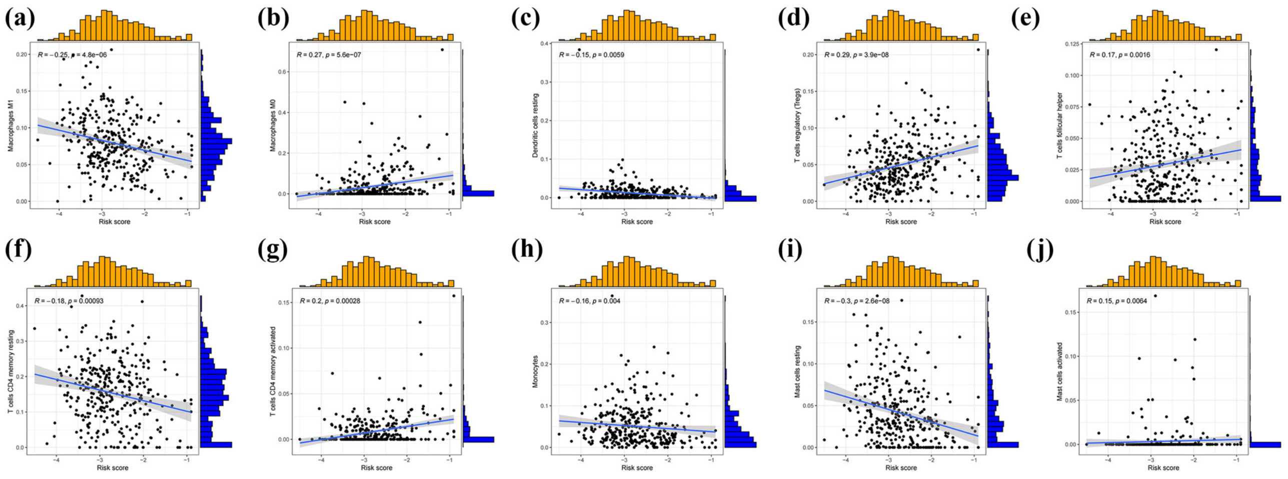

Figure S9: Correlation plot illustrating the correlations between risk scores computed by the hypoxia-glycolysis-related model and immune cells. Scatter plots of the association between risk scores and (a) Macrophages M1, (b) Macrophages M0, (c) Dendritic cells resting, (d) T cells regulatory (Tregs), (e) T cells follicular helper, (f) T cells CD4 memory resting, (g) T cells CD4 memory activated, (h) Monocytes, (i) Mast cells resting, (j) Mast cells activated.

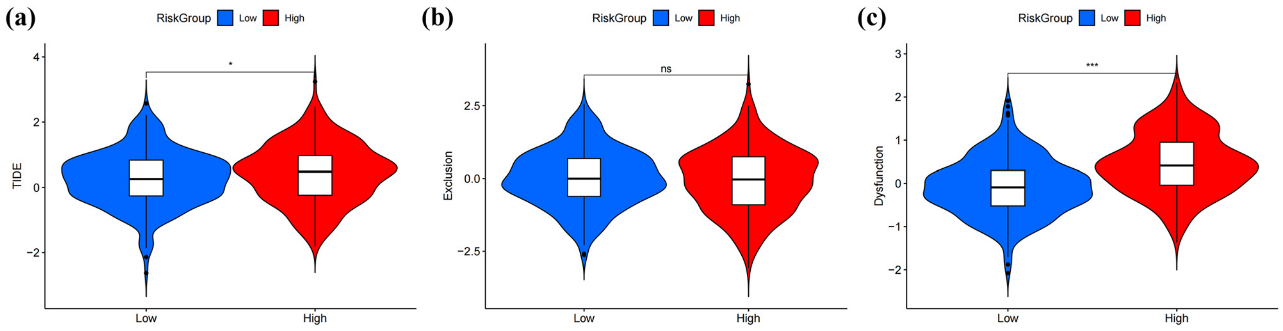

Figure S10: Comparison of TIDE in hypoxia-glycolysis-related risk subgroups. Comparative analysis of (a) TIDE, (b) immune exclusion, and (c) dysfunction between the HG vs the LG. *$p < 0.05$, **$p < 0.01$, ***$p < 0.001$.

Table S1 : Primers for RT-qPCR of 5 HGRGs

Gene id	Primer F	Primer R
ADORA2B	TGCACTGACTTCTACGGCTG	GGTCCCCGTGACCAAACTT
TGFA	AGGTCCGAAAACACTGTGAGT	AGCAAGCGGTTCTTCCCTTC
FBP1	CGCGCACCTCTATGGCATT	TTCTTCTGACACGAGAACACAC
HK3	GGACAGGAGCACCCTCATTTC	CCTCCGAATGGCATCTCTCAG
PDHB	AAGAGGCGCTTTCACTGGAC	ACTAACCTTGTATGCCCCATCA

Table S2: Differentially expressed HGRGs

Gene	log2 Fold Change	P-adjust
ADH1A	−1.860520481	1.42×10^{-15}
ADH1B	−2.597525843	3.30×10^{-22}
ADH1C	−4.338842381	3.31×10^{-48}
ADH6	−2.640574846	5.39×10^{-37}
ADH7	2.937431363	1.92×10^{-8}
ADORA2B	1.00193121	2.18×10^{-13}
AK3	−1.468965107	6.06×10^{-94}
ALDH1A3	−1.284630496	8.98×10^{-12}
ALDH1B1	−1.285761433	4.18×10^{-21}
ALDH3B2	−3.870590253	1.69×10^{-33}
ALDOB	−4.739554118	2.25×10^{-48}
ALDOC	2.226347052	5.31×10^{-50}
BIK	−1.296035268	2.47×10^{-11}
COL5A1	2.041798724	1.94×10^{-28}
CXCR4	2.77023304	1.97×10^{-143}
DCN	−2.543209219	8.87×10^{-25}
DDIT4	2.105637742	4.61×10^{-64}
DPYSL4	2.301387943	4.53×10^{-17}
EFNA3	2.442920842	3.01×10^{-72}
EGFR	1.461954859	1.44×10^{-38}
EGLN3	4.241380452	1.04×10^{-224}
ENO2	3.194700447	3.57×10^{-133}
FBP1	−2.417999959	1.91×10^{-51}
FBP2	−1.313194585	2.82×10^{-8}
G6PC2	1.264762595	8.66×10^{-7}
GALM	−1.195309906	1.88×10^{-45}

(*Continued*)

Table S2: *Continued*

Gene	log2 Fold Change	P-adjust
GAPDH	1.325611166	9.73×10^{-57}
GAPDHS	1.496564764	0.000217537
GPC3	−3.638418038	1.73×10^{-61}
HK2	3.424336741	4.11×10^{-142}
HK3	2.586831498	3.57×10^{-80}
ISG20	2.361234614	1.90×10^{-88}
LDHC	−1.499741299	5.76×10^{-9}
NOL3	3.397413207	2.40×10^{-213}
P4HA1	1.704242983	8.53×10^{-89}
P4HA2	1.25617069	3.55×10^{-34}
PCK1	−2.865209998	1.89×10^{-27}
PCK2	−2.31336016	2.99×10^{-82}
PDHA1	−1.1455	5.52×10^{-48}
PDHB	−1.196107049	9.89×10^{-73}
PFKFB3	−1.262425899	1.41×10^{-27}
PGK2	1.054186175	0.011216359
PPFIA4	3.14638669	3.96×10^{-70}
SAP30	2.471565124	4.18×10^{-176}
SDC3	1.065529445	1.69×10^{-33}
SLC2A1	1.995212771	4.02×10^{-58}
SLC2A3	2.015915749	2.66×10^{-46}
SLC2A5	1.714566364	4.65×10^{-19}
STC2	4.239291296	3.78×10^{-195}
TGFA	1.659360109	2.55×10^{-34}
TGFBI	4.129601139	3.03×10^{-63}
VEGFA	3.480523161	1.32×10^{-180}

Table S3: 25 HGRGs of prognostic significance

Gene	HR	Low95%HR	High95%HR	*P*-value
ADH6	1.4141782	1.165935989	1.71527415	0.000433431
ADORA2B	0.6248799	0.510945888	0.76421977	4.69×10^{-6}
AK3	0.8572948	0.80604244	0.911805946	9.81×10^{-7}
ALDH1A3	1.2229536	1.101627372	1.357642002	0.000159601
ALDH1B1	1.3119434	1.080465574	1.593012776	0.006118259
ALDOB	0.8431662	0.746438238	0.952428851	0.006070675
ALDOC	0.9030009	0.830947862	0.981301761	0.016179278
COL5A1	1.1773746	1.023916739	1.353831779	0.021924359
EFNA3	2.3245175	1.083258153	4.988083155	0.030368457
EGFR	1.4969202	1.207065173	1.856378751	0.000238964
EGLN3	0.8458858	0.779682607	0.917710352	5.69×10^{-5}
ENO2	1.1073452	1.037792314	1.181559511	0.002064683
FBP1	0.7422451	0.650637687	0.846750411	9.20×10^{-6}
G6PC2	0.6337311	0.515554449	0.778996606	1.48×10^{-5}
GALM	0.7359462	0.630492089	0.859038245	0.000102109
GAPDHS	1.2479134	1.084791661	1.435564108	0.001943827
HK3	0.7473295	0.679249855	0.822232682	2.28×10^{-9}
ISG20	0.8973228	0.817505073	0.984933666	0.022645866
PCK1	1.4498354	1.224227339	1.717019842	1.68×10^{-5}
PCK2	0.830201	0.77771504	0.886229014	2.34×10^{-8}
PDHA1	0.6833414	0.571427523	0.817173646	3.01×10^{-5}
PDHB	0.6352852	0.479488021	0.841704626	0.001575446
PGK2	0.5218147	0.390164494	0.697886574	1.16×10^{-5}
TGFA	25.814947	1.982550539	336.1384548	0.013042936
TGFBI	0.5341507	0.29535885	0.966001083	0.038043126

Abbreviation: HR: Hazard Ratio.

Table S4: Five HGRGs for the construction of prognostic model

Gene	Coef	HR	Low95%HR	High95%HR	*P*-value
ADORA2B	0.2164	1.2416	1.0228	1.5072	0.028694
TGFA	−0.19359	0.824	0.7454	0.9109	0.000154
FBP1	−0.24695	0.7812	0.6962	0.8766	2.65×10^{-5}
HK3	0.40217	1.4951	1.2591	1.7753	4.46×10^{-6}
PDHB	−0.39773	0.6718	0.4955	0.911	0.010461

Abbreviation: HR: Hazard Ratio.

Table S5: GSEA pathways for different risk groups

Pathways	Group
**h.all.v2024.1.Hs.symbols.gmt**	
HALLMARK_FATTY_ACID_METABOLISM	Low risk
HALLMARK_PEROXISOME	Low risk
HALLMARK_ADIPOGENESIS	Low risk
HALLMARK_ANDROGEN_RESPONSE	Low risk
HALLMARK_BILE_ACID_METABOLISM	Low risk
**C2.cp.wikipathways.v2024.1.Hs.symbols.gmt**	
WP_10Q22Q23_COPY_NUMBER_VARIATION	Low risk
WP_CHOLESTEROL_BIOSYNTHESIS_PATHWAY_IN_HEPATOCYTES	Low risk
WP_FATTY_ACIDS_AND_LIPOPROTEINS_TRANSPORT_IN_HEPATOCYTES	Low risk
WP_PROXIMAL_TUBULE_TRANSPORT	Low risk
WP_SMITHMAGENIS_AND_POTOCKILUPSKI_SYNDROME_COPY_NUMBER_VARIATION	Low risk
WP_CYTOKINES_AND_INFLAMMATORY_RESPONSE	High risk
WP_IMMUNE_INFILTRATION_IN_PANCREATIC_CANCER	High risk
WP_INFLAMMATORY_RESPONSE_PATHWAY	High risk
WP_OVERVIEW_OF_PROINFLAMMATORY_AND_PROFIBROTIC_MEDIATORS	High risk
**C4.cgn.v2024.1.Hs.symbols.gmt**	
GCM_MAPK10	Low risk
GCM_PTK2	Low risk
GNF2_BNIP3L	Low risk
GNF2_RAD23A	Low risk
MORF_THPO	Low risk
**C5.go.bp.v2024.1.Hs.symbols.gmt**	
GOBP_AMIDE_METABOLIC_PROCESS	Low risk
GOBP_CELLULAR_LIPID_CATABOLIC_PROCESS	Low risk
GOBP_CELLULAR_MODIFIED_AMINO_ACID_METABOLIC_PROCESS	Low risk
GOBP_FATTY_ACID_METABOLIC_PROCESS	Low risk

(*Continued*)

Table S5: *Continued*

Pathways	Group
GOBP_LIPID_MODIFICATION	Low risk
**C7.immunesigdb.v2024.1.Hs.symbols.gmt**	
GSE9650_EFFECTOR_VS_EXHAUSTED_CD8_TCELL_DN	Low risk
GSE21670_TGFB_VS_IL6_TREATED_CD4_TCELL_DN	Low risk
GSE22935_WT_VS_MYD88_KO_MACROPHAGE_48H_MBOVIS_BCG_STIM_UP	Low risk
GSE31622_WT_VS_KLF3_KO_BCELL_UP	Low risk
GSE45365_HEALTHY_VS_MCMV_INFECTION_BCELL_IFNAR_KO_DN	Low risk
**C2.cp.biocarta.v2024.1.Hs.symbols.gmt**	
BIOCARTA_AGR_PATHWAY	Low risk
BIOCARTA_BAD_PATHWAY	Low risk
BIOCARTA_CERAMIDE_PATHWAY	Low risk
BIOCARTA_NFAT_PATHWAY	Low risk
BIOCARTA_PPARA_PATHWAY	Low risk
BIOCARTA_CYTOKINE_PATHWAY	High risk
BIOCARTA_DC_PATHWAY	High risk
BIOCARTA_INFLAM_PATHWAY	High risk
BIOCARTA_NKT_PATHWAY	High risk
BIOCARTA_STEM_PATHWAY	High risk

Abbreviation: GSEA: Gene Set Enrichment Analysis.

Table S6: Antineoplastic drug sensitivity (sensitive group: high)

Drugs	Low-risk group IC50 (25–75%)	High-risk group IC50 (25–75%)	*P*-value
**DNA replication**			
Camptothecin	0.1 (0.07, 0.15)	0.07 (0.05, 0.1)	2.99×10^{-14}
Cisplatin	28.77 (20.6, 42.82)	19.98 (12.52, 29.7)	1.18×10^{-13}
Irinotecan	16.06 (10.83, 22.4)	10.06 (6.73, 15.43)	1.18×10^{-15}
Gemcitabine	0.62 (0.34, 1.11)	0.36 (0.21, 0.62)	2.65×10^{-12}
Temozolomide	400.92 (310.51, 528.87)	352.84 (268.75, 501.15)	0.007870706
Epirubicin	0.38 (0.29, 0.54)	0.31 (0.2, 0.43)	9.61×10^{-9}
Topotecan	1.38 (0.94, 1.99)	0.83 (0.59, 1.26)	1.07×10^{-19}
Teniposide	1.76 (1.17, 2.74)	1.37 (0.83, 2.22)	6.08×10^{-6}
Mitoxantrone	2.03 (1.46, 3.16)	1.47 (0.93, 2.32)	2.78×10^{-10}
Fludarabine	160.33 (130.18, 208.28)	137.92 (103.88, 191.09)	0.000188534
Pyridostatin	30.82 (23.76, 38.97)	24.89 (19.26, 34.07)	9.87×10^{-8}
**Mitosis**			
Vinblastine	0.02 (0.02, 0.04)	0.02 (0.01, 0.03)	0.000136042
Docetaxel	0.01 (0.01, 0.01)	0.01 (0.01, 0.01)	1.90×10^{-5}
Alisertib	6.97 (4.87, 10.18)	5.47 (3.55, 8.93)	1.19×10^{-5}
Paclitaxel	0.06 (0.04, 0.11)	0.05 (0.03, 0.09)	0.007977623
Vinorelbine	0.05 (0.03, 0.08)	0.03 (0.02, 0.06)	2.64×10^{-8}
**Chromatin histone acetylation**			
Vorinostat	4.15 (3.67, 5.11)	4.03 (3.2, 4.87)	0.007367646
Entinostat	10.12 (7.91, 12.54)	7.89 (5.93, 9.73)	1.54×10^{-14}
**Genome integrity**			
Olaparib	75.9 (59.4, 97.38)	63.02 (47.78, 90.7)	1.95×10^{-5}
Mirin	123.96 (98.83, 156.91)	97.6 (80.14, 127.49)	2.06×10^{-11}
Niraparib	76.24 (57.96, 104.59)	64.35 (47.56, 93.93)	0.000112506
Talazoparib	24.68 (18.6, 36.57)	20.51 (13.73, 30.63)	1.80×10^{-5}
VE-822	30.87 (23.97, 39.66)	23.74 (18.29, 31.85)	3.60×10^{-9}
AZD6738	8.54 (6.3, 11.03)	6.16 (4.58, 8.49)	4.39×10^{-11}
Telomerase Inhibitor IX	1.58 (1.3, 2.04)	1.46 (1.12, 1.94)	0.002240436
VE821	59.18 (44.08, 83.38)	52.23 (34.78, 84.89)	0.015098374
**Cell cycle**			
AZD7762	1.28 (0.92, 1.72)	0.85 (0.62, 1.22)	5.43×10^{-18}
Palbociclib	38.16 (29.97, 52.59)	34.08 (25.13, 45.74)	0.000658679
AZD5438	9.06 (7.23, 11.15)	7.95 (6.39, 10.74)	0.002580903
Ribociclib	45.96 (41.4, 50.59)	43.66 (39.38, 49.48)	0.0056315
CDK9_5038	0.1 (0.08, 0.14)	0.08 (0.06, 0.12)	2.26×10^{-7}
MK-8776	26.84 (20.26, 35.35)	20.59 (14.3, 28)	4.28×10^{-10}
**RTK signaling**			
Staurosporine	0.05 (0.04, 0.07)	0.04 (0.03, 0.05)	1.34×10^{-8}

(*Continued*)

Table S6: *Continued*

Drugs	Low-risk group IC50 (25–75%)	High-risk group IC50 (25–75%)	*P*-value
Dasatinib	5.04 (3.67, 7.08)	4.63 (3.07, 6.66)	0.008851312
Foretinib	2.67 (2.22, 3.45)	2.32 (1.87, 3.09)	1.20×10^{-6}
**p53 pathway**			
Nutlin-3a (-)	116.74 (82.29, 154.2)	87.37 (64.9, 138.99)	8.16×10^{-6}
PRIMA-1MET	107.7 (84.25, 136.61)	83.63 (62.42, 108.34)	5.27×10^{-11}
MIRA-1	232.51 (180.99, 298.25)	203.89 (149.88, 292.96)	0.03163603
**ERK MAPK signaling**			
PLX-4720	87.26 (73.65, 108.34)	78.14 (63.35, 100.17)	0.000172587
PD0325901	1.66 (1.32, 2.2)	1.44 (1.12, 2)	0.000327833
Dabrafenib	112.75 (88.38, 132)	89.9 (66.29, 116.74)	2.10×10^{-11}
SCH772984	13.76 (11.38, 17.47)	13.14 (10.35, 16.63)	0.038089895
ERK_2440	15.49 (12.88, 18.51)	13.38 (9.69, 16.89)	1.86×10^{-7}
ERK_6604	34.44 (29.19, 43.09)	27.01 (20.33, 35.19)	3.70×10^{-17}
Selumetinib	72.74 (55.72, 92.91)	58.86 (40.62, 79.77)	5.52×10^{-8}
VX-11e	17.09 (14.36, 20.7)	15.96 (12.26, 19.61)	0.000913905
**Apoptosis regulation**			
Obatoclax Mesylate	3.98 (3.59, 4.8)	3.75 (3.16, 4.44)	1.46×10^{-5}
AZD5582	9.4 (6.41, 15.25)	7.38 (4.43, 12.8)	6.53×10^{-5}
Sabutoclax	0.72 (0.59, 0.87)	0.6 (0.5, 0.76)	2.10×10^{-8}
Venetoclax	8.74 (7.59, 10.31)	7.9 (6.71, 9.82)	0.001209222
ABT737	9.99 (7.42, 12.39)	8.11 (5.62, 11.21)	2.36×10^{-5}
**Hormone-related**			
Tamoxifen	35.71 (31.28, 40.2)	33.53 (29.84, 39.86)	0.016554919
Fulvestrant	18.7 (15.56, 22.47)	17.21 (14.15, 21.41)	0.006593017
**Chromatin histone methylation**			
EPZ004777	185.66 (148.73, 222.96)	151.1 (121.48, 204.2)	2.04×10^{-7}
GSK343	16.95 (14.57, 19.46)	15.66 (13.16, 19.12)	0.009227134
GSK591	99.08 (80.63, 124.05)	90.02 (73.98, 110.64)	0.000476137
**WNT signaling**			
XAV939	87.45 (76.75, 98.24)	74.33 (64.66, 84.81)	3.15×10^{-16}
IWP-2	16.08 (13.64, 19.24)	15.04 (12.53, 19.19)	0.041716291
WIKI4	42.27 (38.52, 46.77)	38.88 (35.18, 42.37)	5.63×10^{-11}
**IGF1R signaling**			
Linsitinib	43.75 (36.34, 55.2)	39.96 (31.62, 52.76)	0.003040346
IGF1R_3801	5.24 (4.01, 7.14)	4.45 (3.16, 6.74)	0.000356893
**PI3K/MTOR signaling**			
Dactolisib	0.19 (0.15, 0.25)	0.18 (0.15, 0.23)	0.046312403
AZD8055	0.82 (0.77, 0.86)	0.8 (0.76, 0.86)	0.025127766
PF-4708671	48.5 (41.22, 58)	45.63 (39.76, 54.23)	0.034491599

(*Continued*)

Table S6: *Continued*

Drugs	Low-risk group IC50 (25–75%)	High-risk group IC50 (25–75%)	*P*-value
Luminespib	0.1 (0.07, 0.16)	0.08 (0.05, 0.14)	0.000143763
CZC24832	160.17 (134, 194.74)	149.13 (116.59, 183.64)	0.000875604
GNE-317	1.69 (1.42, 2.04)	1.56 (1.27, 1.97)	0.002812488
AMG-319	127 (106.3, 167.75)	109.55 (87.88, 148.59)	1.89×10^{-5}
Uprosertib	16.76 (12.57, 22.42)	14.87 (10.78, 20.84)	0.00522278
AZD6482	25.1 (22.23, 28.07)	23.73 (20.86, 27.71)	0.018812359
**Other**			
Cytarabine	5.58 (3.76, 8.48)	4.62 (2.94, 8.31)	0.014020077
5-Fluorouracil	115.67 (79.44, 194.16)	87.04 (58.32, 148.53)	4.78×10^{-6}
AZ960	8.4 (6.11, 11.63)	6.16 (4.45, 9.04)	8.57×10^{-10}
Pevonedistat	1.87 (1.28, 2.77)	1.57 (1.12, 2.84)	0.032734383
WZ4003	40.99 (36.18, 51.21)	37.18 (30.96, 45.49)	$4.22 \times 10^{-}+$
OTX015	11.83 (8.99, 15.97)	10.58 (7.56, 15.7)	0.021015852
Entospletinib	40.42 (33.27, 50.13)	38.06 (30.42, 49.03)	0.037616794
PRT062607	27.98 (23.11, 33.26)	22.71 (19.12, 27.54)	2.19×10^{-12}
IRAK4_4710	142.58 (125.62, 159.41)	133.69 (114.56, 154.07)	0.000854391
JAK1_8709	63.67 (55.03, 73.93)	60.03 (50.12, 75.79)	0.023932807
ULK1_4989	11.31 (8.53, 15.39)	7.85 (5.74, 12.33)	1.67×10^{-11}
VSP34_8731	10.87 (9.21, 12.94)	9.83 (8.22, 12)	0.000186414
JAK_8517	20.7 (15.91, 28.11)	16.9 (12.57, 25.23)	1.72×10^{-5}
GSK2578215A	140.84 (121.94, 159.83)	127.95 (106.9, 150.35)	4.09×10^{-5}
BPD-00008900	88.7 (73.36, 114.67)	80.61 (64.16, 113.24)	0.013887435

Abbreviation: IC50: Half maximal inhibitory concentration.

Table S7: Antineoplastic drug sensitivity (sensitive group: low)

Drugs	Low-risk group IC50 (25–75%)	High-risk group IC50 (25–75%)	*P*-value
**EGFR signaling**			
Gefitinib	24.32 (20.85, 28.04)	26.61 (22.24, 32.45)	3.95×10^{-5}
Afatinib	5.67 (4.81, 7.08)	6.42 (5.18, 7.95)	5.42×10^{-6}
Erlotinib	12.94 (11.17, 15.21)	14.03 (11.18, 17.24)	0.000385006
Sapitinib	50.94 (39.89, 60.22)	54.33 (46.22, 66.37)	0.000245628
AZD3759	13.43 (11.66, 15.86)	15.31 (12.94, 17.98)	3.73×10^{-9}
Osimertinib	4.71 (3.63, 6.2)	5.98 (4.32, 9.24)	3.11×10^{-10}
**Apoptosis regulation**			
LCL161	127 (106.49, 153.96)	137.48 (113.4, 183.84)	0.000143763
**Metabolism**			
GSK2606414	35.78 (29.13, 48.73)	41.56 (30.84, 60.82)	0.000214347
**Chromatin histone acetylation**			
PCI-34051	83.92 (67.47, 107.38)	88.74 (70.07, 122.71)	0.024289601
OF-1	54.16 (47.09, 65.03)	65.95 (53.53, 82.87)	1.25×10^{-12}
**Protein stability and degradation**			
ML323	76.85 (66.69, 96.39)	89.44 (73.8, 117.46)	2.25×10^{-6}
P22077	76.7 (60.21, 97.07)	85.92 (65.4, 135.24)	2.25×10^{-5}
**RTK signaling**			
PD173074	51.87 (37.34, 73.61)	53.71 (38.94, 92.17)	0.048733361
SB505124	9.18 (8.18, 10.03)	10.14 (9, 11.44)	4.54×10^{-12}
AZD4547	16.26 (12.92, 20.24)	17.74 (13.78, 25.89)	0.001416907
Cediranib	7.28 (6.04, 9.09)	8.4 (6.45, 12.41)	4.86×10^{-6}
**Other**			
BMS-345541	22.56 (17.9, 32.58)	27.32 (19.92, 46.54)	3.54×10^{-5}
TAF1_5496	46.24 (37.67, 54.62)	49.31 (38.27, 61.89)	0.002537686
Ibrutinib	75.18 (55.53, 100.66)	98.88 (72.1, 144.12)	9.50×10^{-12}
Zoledronate	40.53 (31.82, 50.76)	42.44 (33.73, 58.65)	0.01294812
Dihydrorotenone	2.22 (1.88, 2.71)	2.62 (2.16, 3.28)	2.13×10^{-10}
Gallibiscoquinazole	11.96 (10.41, 14.98)	13.22 (10.96, 18.02)	0.000118688
Sinularin	31.79 (25.87, 39.62)	36.76 (29.64, 49.22)	3.71×10^{-8}
LY2109761	157.3 (127.2, 196.62)	172.65 (134.04, 249.23)	0.00020632
**Cell cycle**			
RO-3306	19.82 (18.3, 21.1)	20.25 (18.84, 22.16)	0.001683017
BI-2536	1.23 (0.9, 1.58)	1.4 (0.96, 1.88)	0.003863407

Abbreviation: IC50: Half maximal inhibitory concentration.

Table S8: Not obviously sensitive antineoplastic drug information

Drugs	Low-risk group IC50 (25–75%)	High-risk group IC50 (25–75%)	*P*-value
Navitoclax	6.47 (4.6, 9.9)	6.3 (4.07, 10.03)	0.663623479
Nilotinib	35.76 (27.96, 48.7)	33.69 (24.18, 50.48)	0.471918793
Axitinib	21.48 (18.64, 25.04)	21 (17.7, 26.72)	0.60130495
SB216763	192.63 (167.73, 219.8)	183.77 (163.42, 216.58)	0.162270287
KU-55933	78.05 (67.24, 90.3)	75.83 (61.77, 91.53)	0.152255467
NU7441	13.44 (12.02, 14.77)	13.31 (12, 14.95)	0.876355237
Doramapimod	90.68 (82.2, 99.32)	87.3 (79.13, 99.01)	0.187559849
Wee1 Inhibitor	7.13 (5.26, 9.65)	6.56 (4.62, 10.69)	0.305969271
ZM447439	18.85 (16.2, 22.06)	18.12 (15.29, 21.55)	0.071731386
MK-2206	19.61 (15.7, 25.09)	19.2 (15.2, 24.99)	0.838395053
Pictilisib	3.83 (3.15, 4.92)	3.75 (3.02, 4.78)	0.288828917
Crizotinib	24.53 (19.87, 32.67)	22.83 (17.34, 31.85)	0.090941009
Rapamycin	0.11 (0.08, 0.16)	0.11 (0.08, 0.15)	0.583996823
Sorafenib	13.99 (11.55, 17.01)	13.3 (10.51, 18.15)	0.321266533
Oxaliplatin	42.2 (31.54, 55.3)	39.86 (27.99, 56.38)	0.176204428
BMS-536924	7.86 (6.57, 9.67)	7.9 (6.28, 9.94)	0.573104753
MK-1775	1.64 (1.18, 2.2)	1.53 (1.11, 2.43)	0.373451197
Dinaciclib	0.06 (0.05, 0.07)	0.06 (0.04, 0.08)	0.786770328
Bortezomib	0.01 (0.01, 0.01)	0.01 (0.01, 0.01)	0.060433273
GSK269962A	18.36 (15.95, 21.01)	17.11 (15.32, 20.43)	0.050921142
YK-4-279	8.49 (5.96, 13.68)	8.15 (5.24, 14.43)	0.148543729
Trametinib	1.76 (1.35, 2.42)	1.68 (1.2, 2.33)	0.136170185
IAP_5620	155.11 (126.14, 203.85)	165.26 (126.54, 235.03)	0.076238054
AZD2014	7.72 (6.52, 9.52)	7.82 (6.41, 9)	0.360160927
AZD1208	193.85 (167.78, 227.46)	196.69 (160.79, 244.56)	0.478602743
AZD1332	45.65 (37.27, 59.11)	43.54 (34.36, 58.27)	0.112274002
Ruxolitinib	118.26 (97.47, 151.73)	120.88 (91.45, 177.14)	0.55771394
Cyclophosphamide	168 (143.24, 204.63)	164.54 (131.98, 215.71)	0.430914755
Lapatinib	19.42 (16.31, 24.45)	19.63 (15.34, 24.41)	0.337341234
Alpelisib	36.3 (23.92, 51.98)	31.71 (21.53, 51.75)	0.246874553
Taselisib	7.13 (4.67, 11.77)	7.24 (5.08, 12.16)	0.502579844
EPZ5676	252.27 (210.81, 304.47)	240.04 (196.47, 307.14)	0.16904157
OSI-027	117.43 (98.51, 134.15)	117.39 (98.13, 148.68)	0.217550536
LGK974	54.75 (45.47, 65.36)	54.01 (44.59, 70.96)	0.901526342
PFI3	186.49 (158.81, 214.57)	180.53 (151.4, 230.47)	0.828627339
I-BET-762	25.71 (20.75, 31.71)	26.65 (20.84, 35.33)	0.210835228
RVX-208	114.72 (96.88, 134.9)	108.85 (88.31, 146.06)	0.305969271
AGI-6780	59.82 (50.87, 70.05)	60.7 (50.79, 73.24)	0.223335104
Picolinici acid	167.67 (145.92, 193.85)	158.58 (141.14, 196.24)	0.147905323

(*Continued*)

Table S8: *Continued*

Drugs	Low-risk group IC50 (25–75%)	High-risk group IC50 (25–75%)	*P*-value
AZD5153	5.35 (4.27, 6.82)	5.06 (3.72, 7.06)	0.065424249
CDK9_5576	0.65 (0.51, 0.82)	0.62 (0.48, 0.88)	0.519033434
Eg5_9814	0.04 (0.03, 0.06)	0.04 (0.03, 0.07)	0.891624444
AZD5991	69.17 (43.98, 111.86)	73.13 (42.16, 129.18)	0.339350367
PAK_5339	10.47 (8.99, 12.4)	10.27 (8.9, 13.36)	0.441304812
Oxaliplatin	149.87 (118.74, 190.11)	140.73 (105.24, 196.56)	0.209592982
Dactinomycin	0.08 (0.07, 0.1)	0.08 (0.06, 0.11)	0.633352364
Nelarabine	409.66 (323.34, 500.03)	412.67 (313.19, 554.17)	0.439619354
Fulvestrant	88.66 (75.74, 107.36)	93.43 (74.71, 116.73)	0.109220216
Vincristine	0.15 (0.09, 0.28)	0.14 (0.07, 0.3)	0.211666346
Docetaxel	0.08 (0.06, 0.15)	0.08 (0.05, 0.17)	0.205286872
MN-64	111.15 (93.8, 130.92)	108.36 (87.3, 137.12)	0.686905491
KRAS (G12C) Inhibitor-12	74.42 (57.31, 99.83)	76.83 (58.44, 109.48)	0.239069217
MG-132	0.2 (0.18, 0.22)	0.19 (0.17, 0.22)	0.061137569
BDP-00009066	9.95 (8.53, 12.46)	9.65 (7.9, 12.55)	0.170989219
Buparlisib	2.47 (2.11, 2.98)	2.43 (2.04, 3.06)	0.558861241
Ulixertinib	14.91 (12.62, 18.82)	15.66 (12.65, 21.06)	0.103792534
Afuresertib	12.35 (9.97, 15.19)	11.68 (9.68, 16.27)	0.516088155
AZD5363	18.93 (14.46, 24.83)	16.96 (13.21, 24.19)	0.061294999
AZD8186	25.27 (20.72, 31.51)	24.01 (19.77, 30.66)	0.232550633
Ipatasertib	32.37 (25.11, 42.29)	31.87 (25.79, 46.84)	0.412509712
GDC0810	129.12 (112.83, 153.24)	134.69 (109.84, 172.59)	0.105251103
I-BRD9	73.56 (57.06, 96.46)	74.37 (56.55, 111.75)	0.352743624
NVP-ADW742	14.17 (11.19, 19.43)	15.43 (11.47, 20.46)	0.143806679
Savolitinib	13.76 (11.6, 16.35)	13.11 (10.76, 16.38)	0.134681152
UMI-77	14.17 (11.1, 18.01)	14.82 (11.37, 19.84)	0.144275106
Sepantronium bromide	0.01 (0.01, 0.02)	0.01 (0.01, 0.02)	0.079596434
MIM1	48.39 (38.55, 59.39)	45.94 (35.46, 63.02)	0.166586873
WEHI 539	33 (27.33, 42.15)	33.19 (26.23, 43.98)	0.630115124
BIBR-1532	137.79 (112.48, 167.21)	136.45 (108.8, 179.39)	0.937198726
Ulixertinib	8.34 (6.66, 11.2)	9 (6.87, 12.35)	0.175296892
BMS-754807	1.35 (0.91, 1.98)	1.2 (0.82, 2.1)	0.313010496
JQ1	9.97 (6.55, 14.76)	8.73 (5.53, 14.73)	0.130868569

Abbreviation: IC50: Half maximal inhibitory concentration.
